# Path-Integral Methods for Analyzing the Effects of Fluctuations in Stochastic Hybrid Neural Networks

**DOI:** 10.1186/s13408-014-0016-z

**Published:** 2015-02-27

**Authors:** Paul C. Bressloff

**Affiliations:** Department of Mathematics, University of Utah, 155 South 1400 East, Salt Lake City, UT 84112 USA

**Keywords:** Path-integrals, Large deviations, Stochastic neural networks, Stochastic hybrid systems

## Abstract

We consider applications of path-integral methods to the analysis of a stochastic hybrid model representing a network of synaptically coupled spiking neuronal populations. The state of each local population is described in terms of two stochastic variables, a continuous synaptic variable and a discrete activity variable. The synaptic variables evolve according to piecewise-deterministic dynamics describing, at the population level, synapses driven by spiking activity. The dynamical equations for the synaptic currents are only valid between jumps in spiking activity, and the latter are described by a jump Markov process whose transition rates depend on the synaptic variables. We assume a separation of time scales between fast spiking dynamics with time constant $\tau_{a}$ and slower synaptic dynamics with time constant *τ*. This naturally introduces a small positive parameter $\epsilon=\tau _{a}/\tau$, which can be used to develop various asymptotic expansions of the corresponding path-integral representation of the stochastic dynamics. First, we derive a variational principle for maximum-likelihood paths of escape from a metastable state (large deviations in the small noise limit $\epsilon\rightarrow0$). We then show how the path integral provides an efficient method for obtaining a diffusion approximation of the hybrid system for small *ϵ*. The resulting Langevin equation can be used to analyze the effects of fluctuations within the basin of attraction of a metastable state, that is, ignoring the effects of large deviations. We illustrate this by using the Langevin approximation to analyze the effects of intrinsic noise on pattern formation in a spatially structured hybrid network. In particular, we show how noise enlarges the parameter regime over which patterns occur, in an analogous fashion to PDEs. Finally, we carry out a $1/\epsilon$-loop expansion of the path integral, and use this to derive corrections to voltage-based mean-field equations, analogous to the modified activity-based equations generated from a neural master equation.

## Introduction

One of the major challenges in neuroscience is developing our understanding of how noise at the molecular and cellular levels affects dynamics and information processing at the macroscopic level of synaptically coupled neuronal populations. It is well known that the spike trains of individual cortical neurons in vivo tend to be very noisy, having interspike interval (ISI) distributions that are close to Poisson [1, 2]. Indeed, one observes trial-to-trial variability in spike trains, even across trials in which external stimuli are identical. On the other hand, neurons are continuously bombarded by thousands of synaptic inputs, many of which are uncorrelated, so that an application of the law of large numbers would suggest that total input fluctuations are small. This would make it difficult to account for the Poisson-like behavior of individual neurons, even when stochastic ion channel fluctuations or random synaptic background activity is taken into account. One paradigm for reconciling these issues is the so-called balanced network [3–5]. In such networks, each neuron is driven by a combination of strong excitation and strong inhibition, which mainly cancel each other out, so that the remaining fluctuations occasionally and irregularly push the neuron over the firing threshold. Even in the absence of any external sources of noise, the resulting deterministic dynamics is chaotic and neural outputs are Poisson-like. Interestingly, there is some experimental evidence that cortical networks can operate in a balanced regime [6].

Another emergent feature of balanced networks is that they can support an asynchronous state characterized by large variability in single neuron spiking, and yet arbitrarily small pairwise correlations, even in the presence of substantial amounts of shared inputs [7]. Thus there is a growing consensus that the trial-to-trial irregularity in the spiking of individual neurons is often unimportant, and that information is typically encoded in firing rates. There is then another level of neural variability, namely, trial-to-trial variations in the firing rates themselves. Recent physiological data shows that the onset of a stimulus reduces firing-rate fluctuations in cortical neurons, while having little or no effect on the spiking variability [8]. Litwin-Kumar and Doiron have recently shown how these two levels of stochastic variability can emerge in a balanced network of randomly connected spiking neurons, in which a small amount of clustered connections induces firing-rate fluctuations superimposed on spontaneous spike fluctuations [9].

Various experimental and computational studies of neural variability thus motivate the incorporation of noise into rate-based neural network models [10]. One approach is to add extrinsic noise terms to deterministic models resulting in a neural Langevin equation [11–15]. An alternative approach is to assume that noise arises intrinsically as a collective population effect, and to describe the stochastic dynamics in terms of a neural master equation [16–20]. In the latter case, neurons are partitioned into a set of *M* local homogeneous populations labeled $\alpha=1,\ldots,M$, each consisting of ${\mathcal{N}}$ neurons. The state of each population at time *t* is specified by the number ${\mathcal{N}}_{\alpha}(t)$ of active neurons in a sliding window $(t,t+\Delta t]$, and transition rates between the discrete states are chosen so that standard rate-based models are obtained in the mean-field limit, where statistical correlations can be ignored. There are two versions of the neural master equation, which can be distinguished by the size of the sliding window width Δ*t*. (Note that the stochastic models are keeping track of *changes* in population activity.) One version assumes that each population operates close to an asynchronous state for large ${\mathcal{N}}$ [18, 19], so that one-step changes in population activity occur relatively slowly. Hence, one can set $\Delta t =1$ and take ${\mathcal{N}}$ to be large but finite. The other version of the neural master equation assumes that population activity is approximately characterized by a Poisson process [17, 20]. In order to maintain a one-step jump Markov process, it is necessary to take the limits $\Delta t \rightarrow0$, ${\mathcal{N}}\rightarrow\infty$ such that ${\mathcal{N}}\Delta t=1$. Thus, one considers the number of active neurons in an infinite background sea of inactive neurons, which is reasonable if the networks are in low activity states. (Note that it is also possible to interpret the master equation of Buice et al. in terms of activity states of individual neurons rather than populations [17, 20].)

One way to link the two versions of the neural master equation is to extend the Doi–Peliti path-integral representation of chemical master equations [21–23] to the neural case; the difference between the two versions then reduces to a different choice of scaling of the underlying action functional [18]. Buice et al. [17, 20] used diagrammatic perturbations methods (Feynman graphs) to generate a truncated moment hierarchy based on factorial moments, and thus determined corrections to mean-field theory involving coupling to two-point and higher-order cumulants. They also used renormalization group methods to derive scaling laws for statistical correlations close to criticality, that is, close to a bifurcation point of the underlying deterministic model [17]. On the other hand, Bressloff [18, 19] showed how the path-integral representation of the master equation can be used to investigate large deviations or rare event statistics underlying escape from the basin of attraction of a metastable state, following along analogous lines to previous work on large deviations in chemical master equations [24–26].

One limitation of both versions of the neural master equation is that they neglect the dynamics of synaptic currents. The latter could be particularly significant if the time scale *τ* of synaptic dynamics is larger than the window width Δ*t*. Therefore, we recently extended the Buice et al. neural master equation by formulating the network population dynamics in terms of a stochastic hybrid system also known as a ‘velocity’ jump Markov process [27]. The state of each population is now described in terms of two stochastic variables $U_{\alpha}(t)$ and ${\mathcal{N}}_{\alpha}(t)$. The synaptic variables $U_{\alpha}(t)$ evolve according to piecewise-deterministic dynamics describing, at the population level, synapses driven by spiking activity. These equations are only valid between jumps in spiking activity ${\mathcal{N}}_{\alpha}(t)$, which are described by a jump Markov process whose transition rates depend on the synaptic variables. We also showed how asymptotic methods recently developed to study metastability in other stochastic hybrid systems, such as stochastic ion channels, motor-driven intracellular cargo transport, and gene networks [28–32], can be extended to analyze metastability in stochastic hybrid neural networks, in a regime where the synaptic dynamics is much slower than the spiking dynamics. In the case of ion channels, ${\mathcal{N}}_{\alpha }$ would represent the number of open channels of type *α*, whereas $U_{\alpha}$ would be replaced by the membrane voltage *V*. On the other hand, for intracellular transport, ${\mathcal{N}}_{\alpha}$ would be the number of motors of type *α* actively transporting a cargo and $U_{\alpha}$ would be replaced by spatial position along the track.

In this paper we show how a path-integral representation of a stochastic hybrid neural network provides a unifying framework for a variety of asymptotic perturbation methods. The basic hybrid neural network model is described in Sect. 2, where we consider several limiting cases. In Sect. 3, we reprise the path-integral construction of Bressloff and Newby [33], highlighting certain features that were not covered in the original treatment, including the connection with large-deviation principles [34], and potential difficulties in the thermodynamic limit ${\mathcal{N}}\rightarrow\infty$. In Sect. 4, we derive the basic variational principle that can be used to explore maximum-likelihood paths of escape from a metastable state, and relate the theory to the underlying Hamiltonian structure of the path-integral representation. In Sect. 5, we show how the path-integral representation provides an efficient method for deriving a diffusion approximation of a stochastic hybrid neural network. Although the diffusion approximation breaks down when considering escape problems, it provides useful insights into the effects of fluctuations within the basin of attraction of a given solution. We illustrate this by using the diffusion approximation to explore the effects of noise on neural pattern formation in a spatially structured network. In particular, we show how noise expands the parameter regime over which patterns can be observed, in an analogous fashion to stochastic PDEs. Finally, in Sect. 6, we use the path-integral representation to derive corrections to voltage-based mean-field equations, along analogous lines to the analysis of activity-based mean-field equations arising from the neural master equation [17, 20].

## Stochastic Hybrid Network Model

We first describe a stochastic neural network model that generalizes the neural master equation [17, 18, 20] by incorporating synaptic dynamics. (A more detailed derivation of the model can be found in [27].) Note that there does not currently exist a complete, rigorous derivation of population rate-based models starting from detailed biophysical models of individual neurons, although some significant progress has been made in a series of papers by Buice and Chow on generalized activity equations for theta neurons [35–37]. Therefore, the construction of the stochastic rate-based model is phenomenological in nature. However, it is motivated by the idea that finite-size effects in local populations of neurons acts as a source of intrinsic noise. Consider a set of *M* homogeneous populations labeled $\alpha=1,\ldots ,M$, with ${\mathcal{N}}$ neurons in each population. (A straightforward generalization would be for each population to consist of ${\mathcal{O}}({\mathcal{N}})$ neurons.) The output activity of each population is taken to be a discrete stochastic variable $A_{\alpha}(t)$ given by

2.1 $$ A_{\alpha}(t)=\frac{{\mathcal{N}}_{\alpha}(t)}{{\mathcal{N}}\Delta t}, $$

where ${\mathcal{N}}_{\alpha}(t)$ is the number of neurons in the *α*th population that fired in the time interval $[t-\Delta t,t]$, and Δ*t* is the width of a sliding window that counts spikes. The discrete stochastic variables ${\mathcal{N}}_{\alpha}(t)$ are taken to evolve according to a one-step jump Markov process:

2.2 $$ {\mathcal{N}}_{\alpha}(t) \overset{\omega_{+}/\tau_{a}}{\rightarrow}{\mathcal{N}}_{\alpha}(t) + 1,\quad\quad {\mathcal{N}}_{\alpha}(t) \overset {\omega_{-}/\tau_{a}}{\rightarrow}{\mathcal{N}}_{\alpha}(t)- 1 $$

with corresponding transition rates

2.3 $$ \omega_{+}={{\mathcal{N}}\Delta t} F(U_{\alpha}),\quad\quad \omega_{-} ={{ \mathcal{N}}_{\alpha}}. $$

Here *F* is a sigmoid firing-rate or gain function

2.4 $$ F(u)=\frac{F_{0}}{1+\mathrm {e}^{-\gamma(u-\kappa)}}, $$

where *γ*, *κ* correspond to the gain and threshold, respectively, $F_{0}$ is the maximum firing rate, and $U_{\alpha}(t)$ is the effective synaptic current into the *α*th population, which evolves (for exponential synapses) according to

2.5 $$ \tau\, dU_{\alpha}(t)= \Biggl[-{U_{\alpha}(t)}+\sum _{\beta=1}^{M} w_{{\alpha }\beta}A_{\beta}(t) \Biggr]\,dt. $$

We will assume that ${\mathcal{N}}$ is large but finite and take ${\mathcal{N}}\Delta t=1$. In the dual limits ${\mathcal{N}}\rightarrow \infty$ and $\tau\rightarrow0$, our model then reduces to the Buice et al. [17, 20] version of the neural master equation. The resulting stochastic process defined by (2.1)–(2.5) is an example of a stochastic hybrid system based on a piecewise-deterministic process. That is, the transition rate $\omega _{+}$ depend on $U_{\alpha}$, with the latter itself coupled to the associated jump Markov according to (2.5), which is only defined between jumps, during which $U_{\alpha}(t)$ evolves deterministically. It is important to note that the time constant $\tau _{a}$ cannot be identified directly with membrane or synaptic time constants. Instead, it determines the relaxation rate of a local population to the instantaneous firing rate.

Introduce the probability density

$$\begin{aligned} &\operatorname{Prob}\bigl\{ U_{\alpha}(t)\in(u_{\alpha},u_{\alpha}+du), {\mathcal{N}}_{\alpha}(t)=n_{\alpha};\alpha=1,\ldots,M\bigr\} \\ &\quad=p(\mathbf {u},\mathbf{n},t|\mathbf {u}_{0},\mathbf{n}_{0},0)\,d\mathbf {u}, \end{aligned}$$

with $\mathbf{u}=(u_{1},\ldots,u_{M})$ and $\mathbf{n}=(n_{1},\ldots,n_{M})$. It follows from (2.1)–(2.5) that the probability density evolves according to the differential Chapman–Kolmogorov (CK) equation (dropping the explicit dependence on initial conditions)

2.6 $$\begin{aligned} & \frac{\partial p}{\partial t}+\frac{1}{\tau}\sum _{\alpha}\frac {\partial [v_{\alpha}(\mathbf{u},\mathbf{n})p(\mathbf{u},\mathbf{n},t)]}{\partial u_{\alpha}} \\ &\quad=\frac {1}{\tau_{a}}\sum _{\alpha} ({\mathbb{T}}_{\alpha}-1) \bigl(n_{\alpha }p(\mathbf{u},\mathbf{n},t) \bigr) \\ &\quad\quad{} +\frac{1}{\tau_{a}}\sum_{\alpha} \bigl( {\mathbb{T}}_{\alpha }^{-1}-1\bigr) \bigl(F(u_{\alpha})p(\mathbf{u},\mathbf{n},t) \bigr), \end{aligned}$$

with

2.7 $$ v_{\alpha}(\mathbf{u},\mathbf{n})=-u_{\alpha}+\sum _{\beta}w_{\alpha\beta }n_{\beta}, $$

and ${\mathbb{T}}_{\alpha}$ the translation operator: ${\mathbb{T}}_{\alpha}^{\pm1}f(\mathbf{n})=f(\mathbf{n}_{\alpha\pm})$ for any function *f* with $\mathbf{n}_{\alpha\pm}$ denoting the configuration with $n_{\alpha}$ replaced by $n_{\alpha}\pm1$. Equation (2.6) can be re-expressed in the more general form

2.8 $$ \frac{\partial p}{\partial t} = -\frac{1}{\tau}\sum_{{\alpha }=1}^{M} \frac {\partial}{\partial u_{\alpha}}\bigl(v_{\alpha}(\mathbf{u},\mathbf{n})p(\mathbf{u},\mathbf{n},t)\bigr) + \frac {1}{\tau_{a}}\sum_{\mathbf{m}}W(\mathbf{n},\mathbf{m};\mathbf{u})p(\mathbf{u},\mathbf{m},t). $$

The drift ‘velocities’ $v_{\alpha}(\mathbf{u},\mathbf{n})$ for fixed **n** represent the piecewise-deterministic synaptic dynamics according to

2.9 $$ \tau\frac{du_{\alpha}}{dt}=v_{\alpha}(\mathbf{u},\mathbf{n}),\quad \alpha =1,\ldots,M, $$

and *W* is defined in terms of the **u**-dependent transition matrix *T* for the jump Markov process, that is,

$$W(\mathbf{n},\mathbf{m};\mathbf{u})=T(\mathbf{n},\mathbf{m};\mathbf{u})-\delta_{\mathbf{n},\mathbf{m}}\sum _{{\mathbf{k}} }T({\mathbf{k}},\mathbf{m};\mathbf{u}). $$

It follows from (2.6) that *W* can be written as

$$W(\mathbf{n},\mathbf{m};\mathbf{u})=\sum_{\alpha=1}^{M} W^{\alpha}(n_{\alpha },m_{\alpha};u_{\alpha })\prod _{\beta\neq\alpha}\delta_{n_{\beta},m_{\beta}} $$

with $W^{\alpha}$ the tridiagonal matrix

$$\begin{aligned} W^{\alpha}(n,n-1;u) =&F(u), \quad\quad W^{\alpha}(n,n+1;u)=n+1,\\ W^{\alpha }(n,n;u) =&-F(u)-n. \end{aligned}$$

For fixed *u*, the matrix $W^{\alpha}$ is irreducible (which means that there is a non-zero probability of transitioning, possibly in more than one step, from any state to any other state in the jump Markov process). Moreover, all off-diagonal elements are non-negative. It follows that the full transition matrix $W(\mathbf{n},\mathbf{m};\mathbf{u})$ also has these properties and, hence, we can apply the Perron–Frobenius theorem to show that there exists a unique invariant measure for the Markov process. That is, the master equation

$$\frac{d p(\mathbf{u},\mathbf{n},t)}{d t}=\frac{1}{\tau_{a}}\sum_{\mathbf{m}}W(\mathbf{n}, \mathbf{m};\mathbf{u})p(\mathbf{u},\mathbf{m},t), $$

has a globally attracting steady state $\rho(\mathbf{u},\mathbf{n})$ such that $p(\mathbf{u},\mathbf{n},t)\rightarrow\rho(\mathbf{u},\mathbf{n})$ as $t\rightarrow\infty$.

The Perron–Frobenius theorem states that [38] a real square matrix with positive entries has a unique largest real eigenvalue (the Perron eigenvalue) and that the corresponding eigenvector has strictly positive components. If we define a new transition matrix $\widehat {W}(\mathbf{n},\mathbf{m};\mathbf{u})$ by

$$\widehat{W}(\mathbf{n},\mathbf{m};\mathbf{u})={W}(\mathbf{n},\mathbf{m};\mathbf{u})+\delta_{\mathbf{n},\mathbf{m}}W^{*}, \quad W^{*}= \max_{\mathbf{m}} W(\mathbf{m},\mathbf{m};\mathbf{u})+\kappa, $$

for an arbitrary $\kappa> 0$, then we can apply the Perron–Frobenius theorem directly to $\widehat{W}$ and thus to *W*. Since $\sum_{\mathbf{n}}W(\mathbf{n},\mathbf{m};\mathbf{u})=0$ for all **m**, that is, $\eta(\mathbf{n})=(1,1,\ldots ,1)^{T}$ is a left null-vector, it follows that the Perron eigenvalue is $\lambda=0$. The unique invariant measure then corresponds to the right null-vector of *W* for fixed **u**:

2.10 $$ \sum_{\mathbf{m}}W(\mathbf{n},\mathbf{m};\mathbf{u})\rho(\mathbf{u},\mathbf{m})=0. $$

The steady-state solution $\rho(\mathbf{u},\mathbf{n})$ of (2.6) can be factorized as $\rho(\mathbf{u},\mathbf{n})= \prod_{\beta=1}^{M} \rho_{0}(u_{\beta },n_{\beta})$ with

2.11 $$ 0 =\sum_{\alpha=1}^{M} \biggl[ \prod_{\beta\neq\alpha}\rho_{0}(u_{\beta },n_{\beta}) \biggr] \bigl[J(u_{\alpha},n_{\alpha}+1)-J(u_{\alpha },n_{\alpha}) \bigr], $$

where

$$J(u,n)=n\rho_{0}(u,n)-F(u)\rho_{0}(u,n-1). $$

A sufficient condition for (2.11) to hold is

$$J(u,n+1)-J(u,n)=0. $$

Since $\rho_{0}(u,-1)\equiv0$, it then follows that $J(u,0)=0$ and thus $J(u,n)=0$ for all *n*. Hence, we obtain the positive steady-state solution

2.12 $$ \rho_{0}(u,n)=\rho_{0}(u,0)\prod _{m=1}^{n}\frac{F(u)}{m}=\rho _{0}(u,0) \frac{F(u)^{n}}{n!}. $$

The Perron–Frobenius theorem ensures that this is the unique positive solution. The fact that the steady state factorizes is a consequence of the fact that the transition rates do not involve any coupling between populations—the only coupling appears in the drift terms of (2.6). Strictly speaking, the Perron–Frobenius theorem applies to finite-dimensional matrices, so we are assuming that ${\mathcal{N}}$ is finite. Nevertheless, in the thermodynamic limit ${\mathcal{N}}\rightarrow\infty$, the corresponding normalized density reduces to a Poisson process with rate $F(u)$:

2.13 $$ \rho_{0}(u,n)= \mathrm {e}^{-F(u)}\frac{F(u)^{n}}{n!}. $$

There are two time scales in the CK equation (2.8), the synaptic time constant *τ* and the time constant $\tau_{a}$, which characterizes the relaxation rate of population activity. In the limit $\tau\rightarrow0$, (2.5) reduces to the neural master equation of Buice et al. [17, 20]. First, note that the synaptic variables $U_{\alpha}(t)$ are eliminated by setting $v_{\alpha }=0$, that is, $U_{\alpha}(t)=\sum_{\beta}w_{\alpha\beta}A_{\beta }(t)$. This then leads to a pure birth-death process for the discrete variables ${\mathcal{N}}_{\alpha}(t)$. That is, let $P({\mathbf{n}},t) = \operatorname{Prob}[\boldsymbol {\mathcal{N}}(t) =\mathbf{n}]$ denote the probability that the network of interacting populations has configuration ${\mathbf{n}} = (n_{1},n_{2},\ldots,n_{M})$ at time *t*, $t >0$, given some initial distribution $P({\mathbf{n}},0)$. The probability distribution then evolves according to the birth-death master equation [17, 18, 20]

2.14 $$\begin{aligned} \frac{dP(\mathbf{n},t)}{dt} =& \sum_{\alpha}\bigl[ ({\mathbb{T}}_{\alpha}-1) \bigl(\varOmega_{\alpha}^{-}(\mathbf{n})P(\mathbf{n},t) \bigr) \\ &{}+ \bigl( { \mathbb{T}}_{\alpha }^{-1}-1\bigr) \bigl(\varOmega_{\alpha}^{+}( \mathbf{n})P(\mathbf{n},t) \bigr) \bigr], \end{aligned}$$

where

2.15 $$ \varOmega_{\alpha}^{+}(\mathbf{n})=\frac{1}{\tau_{a}}F \biggl(\sum _{\beta }w_{\alpha \beta}n_{\beta} \biggr),\qquad \varOmega_{\alpha}^{-}(\mathbf{n})=\frac {n_{\alpha }}{\tau_{a}}. $$

Buice et al. [20] show that the network operates in a Poisson-like regime in which the rates of the Poisson process are stochastic variables whose means evolve according to the activity-based mean-field equation

2.16 $$ \tau_{\alpha}\frac{dA_{\alpha}}{dt}=-A_{\alpha}(t)+F\biggl(\sum _{\beta }w_{\alpha\beta}A_{\beta}(t)\biggr) . $$

On the other hand, if $\tau_{a}\rightarrow0$ for fixed *τ*, then we obtain deterministic voltage or current-based mean-field equations

2.17 $$\begin{aligned} \tau\frac{du_{\alpha}}{dt} =& \sum_{\mathbf{n}}v_{\alpha} \bigl(\mathbf{u}(t),\mathbf{n}\bigr)\rho\bigl(\mathbf{u} (t),\mathbf{n}\bigr) \\ =& -{u_{\alpha}(t)}+\sum_{\beta=1}^{M} w_{{\alpha}\beta}\sum_{\mathbf{n}} n_{\beta}\rho\bigl( \mathbf{u}(t),\mathbf{n}\bigr). \end{aligned}$$

Since $\rho(\mathbf{u},\mathbf{n})$ is given by a product of independent Poisson processes with rates $F(u_{\alpha})$, consistent with the operating regime of the Buice et al. master equation [17, 20], it follows that

2.18 $$ \langle n_{\beta}\rangle= F(u_{\beta}), $$

and (2.17) reduces to the standard voltage or current-based activity equation

2.19 $$ \tau\frac{du_{\alpha}}{dt}= -{u_{\alpha}(t)}+\sum _{\beta=1}^{M} w_{{\alpha}\beta}F(u_{\beta}). $$

Note that the limit $\tau_{a}\rightarrow0$ is analogous to the slow synapse approximation used by Ermentrout [39] to reduce deterministic conductance-based neuron models to voltage-based rate models. Now suppose that the network operates in the regime $0<\tau _{a}/\tau\equiv\epsilon\ll1$. There is then a natural small parameter in the system, *ϵ*, which allows a variety of perturbation methods to be used:

(i) A quasi-steady-state (QSS) diffusion approximation of the stochastic hybrid system, in which the CK equation (2.6) reduces to a Fokker–Planck equation [27]. This exploits the fact that for small *ϵ* there are typically a large number of transitions between different firing states **n** while the synaptic currents **u** hardly change at all. This implies that the system rapidly converges to the (quasi) steady state $\rho(\mathbf{u},\mathbf{n})$, which will then be perturbed as **u** slowly evolves.
(ii) The diffusion approximation captures the Gaussian-like fluctuations within the basin of attraction of a fixed point of the mean-field equations. However, for small *ϵ* this yields exponentially large errors for the transition rates between metastable states. (A similar problem arises in approximating chemical and neural master equations by a Fokker–Planck equation in the large *N* limit [19, 24, 40].) However, one can use a Wentzel–Kramers–Brillouin (WKB) approximation of solutions to the full CK equation to calculate the mean first passage time for escape [27].
(iii) Another way to analyze the dynamics of a stochastic hybrid network is to derive moment equations. However, for a nonlinear system, this yields an infinite hierarchy of coupled moment equations, resulting in the problem of moment closure. In the case of small *ϵ*, one can expand the moment equations to some finite order in *ϵ*.

In this paper, we show how a path-integral representation of a stochastic hybrid system provides a unifying framework for carrying out all three perturbation schemes highlighted above.

## Path-Integral Representation

### One-Population Model

We now derive the path-integral representation of a stochastic hybrid neural network using the construction introduced in [33]. For ease of notation, we consider a one-population model ($M=1$); the generalization to multiple populations is then straightforward (see Sect. 3.4). We first discretize time by dividing a given interval $[0,T]$ into *N* equal subintervals of size Δ*t* such that $T=N\Delta t$ and set $u_{j}=u(j\Delta t)$, $n_{j}=n(j\Delta t)$. (Note that the infinitesimal time interval Δ*t* used in path discretization is distinct from the width of the moving window used in the construction of the stochastic neural network; see Sect. 2. One should also take care to distinguish between the discrete time label *j* and the population label *α*.) The conditional probability density for $u_{1},\ldots,u_{N}$, given $u_{0}$ and a particular realization of the stochastic discrete variables $n_{j}$, $j=0,\ldots,N-1$, is

$$P(u_{1},\ldots,u_{N}|u_{0},n_{0}, \ldots, n_{N-1}) =\prod_{j=0}^{N-1} \delta \bigl(u_{j+1}-u_{j}-v_{n_{j}}(u_{j}) \Delta t \bigr), $$

where

3.1 $$ v_{n}(u)\equiv v(u,n)=-u+w n. $$

Inserting the Fourier representation of the Dirac delta function gives

$$\begin{aligned} &P(u_{1},\ldots,u_{N}|u_{0},n_{0},n_{1}, \ldots, n_{N-1}) \\ &\quad=\prod_{j=0}^{N-1} \biggl[\int _{-\infty}^{\infty} \mathrm {e}^{ -ip_{j} (u_{j+1}-u_{j}-v_{n_{j}}(u_{j})\Delta t ) } \frac{ dp_{j}}{ 2\pi} \biggr]. \end{aligned}$$

On averaging with respect to the intermediate states $n_{j}$, $j=1,N-1$, we have

3.2 $$\begin{aligned} & P(u_{1},\ldots,u_{N}|u_{0},n_{0}) \\ & \quad= \Biggl[\prod_{j=0}^{N-1}\int _{-\infty}^{\infty}\frac { dp_{j}}{ 2\pi} \Biggr]\sum _{n_{1},\ldots,n_{N-1}}\prod_{j=0}^{N-1}T_{n_{j+1}, n_{j}}(u_{j}) \mathrm {e}^{ -ip_{j} (u_{j+1}-u_{j}-v_{n_{j}}(u_{j})\Delta t ) }, \end{aligned}$$

where

$$T_{n_{j+1}, n_{j}}(u_{j}) = \biggl(\delta_{n_{j+1}, n_{j}}+W_{n_{j+1, }n_{j}}(u_{j}) \frac{\Delta t}{\epsilon} \biggr)+ o(\Delta t) $$

and $W_{nm}(u)\equiv W(n,m;u)$ such that

3.3 $$ W_{n,n-1}=F(u), \quad\quad W_{nn} =-F(u)-n,\quad\quad W_{n,n+1}=n+1. $$

In order to evaluate the above path integral, we introduce the eigenvalue equation

3.4 $$ \sum_{m} \bigl[W_{nm}(u) + q \delta_{n,m}v_{m}(u) \bigr]R_{m}^{(s)}(u, q) =\lambda_{s}(u, q)R_{n}^{(s)}(u, q), $$

and let $\xi_{m}^{(s)}$ be the adjoint eigenvector satisfying

3.5 $$ \sum_{n}\xi_{n}^{(s)}(u, q) \bigl[W_{nm}(u) + q \delta _{n,m}v_{m}(u) \bigr]= \lambda_{s}(u, q)\xi_{n}^{(s)}(u, q). $$

In our original construction of the path-integral representation [33], we arrived at (3.4) and its adjoint through trial and error, based on our previous work on WKB methods. It turns out that the principal eigenvalue of the linear equation (3.4) can be related to the rate function of large-deviation theory, as we explain in Sect. 3.2. A basic result from linear algebra is that $R^{(s)}$ and $\xi^{(s)}$ form a bi-orthonormal set for fixed *u*, *q*. First, rewrite (3.4) and (3.5) in the compact form

$$L^{\dagger}\xi^{(s)}=\lambda_{s}\xi^{(s)},\quad\quad LR^{(s)}=\lambda_{s}R^{(s)}. $$

Defining the inner product $\langle\xi^{(s)},R^{(s')}\rangle=\sum_{n}\xi _{n}^{(s)}R_{n}^{(s')}$, we see that

$$0=\bigl\langle \xi^{(s)},LR^{(s')}\bigr\rangle -\bigl\langle L^{\dagger}\xi ^{(s)},R^{(s')}\bigr\rangle =( \lambda_{s}-\lambda_{s'})\bigl\langle \xi ^{(s)},R^{(s')}\bigr\rangle . $$

Thus, for distinct eigenvalues ($\lambda_{s}\neq\lambda_{s'}$) the eigenvectors $R^{(s')}$ and $\xi^{(s)}$ are orthogonal, and this can be extended to degenerate eigenvalues by Schmidt orthogonalization. Now suppose that we expand a general vector *v* according to $v=\sum_{s}c_{s}R^{(s)}$ for coefficients $c_{s}$. Biorthogonality implies that $c_{s}=\langle\xi^{(s)},v\rangle$. Substituting back into the eigenvector expansion of *v* gives

$$v_{n}=\sum_{s} \bigl\langle \xi^{(s)},v\bigr\rangle R_{n}^{(s)}=\sum _{s}\sum_{m} \xi_{m}^{(s)} \xi_{m}^{(s)}R_{n}^{(s)}, $$

which leads to the completeness relation

3.6 $$ \sum_{s}\xi_{m}^{(s)}(u, q)R_{n}^{(s)}(u, q)=\delta_{m,n} $$

for all *u*, *q*.

Now suppose that we insert multiple copies of the identity (3.6) into the path integral (3.2) with $q=q_{j}$ at the $(j+1)$th time step. That is, taking

$$\begin{aligned} T_{n_{j+1}n_{j}}(u_{j}) =&\sum_{m}T_{mn_{j}}(u_{j}) \delta_{m,n_{j+1}} \\ =& \sum_{s_{j}, m}R_{n_{j+1}}^{(s_{j})}(u_{j}, q_{j})\xi _{m}^{(s_{j})}(u_{j}, q_{j}) \biggl(\delta_{n_{j}, m}+A_{mn_{j}}(u_{j}) \frac{\Delta t}{\epsilon} \biggr) \\ =&\sum_{s_{j}} \biggl(1+\bigl[\lambda_{s_{j}}(u_{j}, q_{j})-q_{j}v_{n_{j}}(u_{j})\bigr] \frac{\Delta t}{\epsilon} \biggr) R_{n_{j+1}}^{(s_{j})}(u_{j}, q_{j})\xi_{n_{j}}^{(s)}(u_{j}, q_{j}) \\ \sim&\sum_{s_{j}}\exp \biggl(\bigl[ \lambda_{s_{j}}(x_{j}, q_{j}) - q_{j}v_{n_{j}}(x_{j}) \bigr]\frac{\Delta t}{\epsilon} \biggr)R_{n_{j+1}}^{(s_{j})}(x_{j}, q_{j})\xi_{n_{j}}^{(s_{j})}(x_{j}, q_{j}) \end{aligned}$$

we find that

3.7 $$\begin{aligned} & P(u_{1},\ldots,u_{N}|u_{0},n_{0}) \\ &\quad= \Biggl[\prod_{j=0}^{N-1}\int _{-\infty}^{\infty}\frac { dp_{j}}{ 2\pi} \Biggr]\sum _{n_{1},\ldots,n_{N-1}}\sum_{s_{j}}\exp \biggl( \biggl[\lambda_{s_{j}}(u_{j}, q_{j})-i\epsilon p_{j}\frac{u_{j+1}-u_{j}}{\Delta t} \biggr]\frac{\Delta t}{\epsilon } \biggr) \\ &\quad\quad{} \times\exp \biggl(\bigl[i\epsilon p_{j}v_{n_{j}}(u_{j})-q_{j}v_{n_{j}}(u_{j}) \bigr]\frac{\Delta t}{\epsilon} \biggr) R_{n_{j+1}}^{(s_{j})}(u_{j}, q_{j})\xi_{n_{j}}^{(s_{j})}(u_{j}, q_{j}), \end{aligned}$$

to leading order in $O(\Delta u,\Delta t)$. It is important to note that the total path integral is independent of the $q_{j}$, since performing the summations over $s_{j}$ recovers the Kronecker deltas. Let us now introduce the probability density

3.8 $$ P(u_{N},n_{N}|u_{0},n_{0})= \Biggl[ \prod_{j=1}^{N-1} \int_{-\infty}^{\infty }\,du_{j} \Biggr]P(u_{1},\ldots,u_{N},n_{N}|u_{0},n_{0}). $$

Substituting for *P* using (3.2) and (3.7), leads to

$$\begin{aligned} &P(u_{N},n_{N}|u_{0},n_{0}) \\ &\quad= \Biggl[\prod_{j=1}^{N-1} \int _{-\infty}^{\infty}du_{j} \Biggr] \Biggl[\prod _{j=0}^{N-1} \int_{-\infty}^{\infty} \frac{dp_{j}}{2\pi} \Biggr] \\ &\quad\quad{}\times\sum_{n_{1},\ldots,n_{N-1}}\sum _{s_{0},\ldots,s_{N-1}}\Biggl[\prod_{j=0}^{N-1} R_{n_{j+1}}^{(s_{j})}(u_{j}, q_{j})\xi _{n_{j}}^{(s_{j})}(u_{j}, q_{j}) \Biggr] \\ &\quad\quad{}\times\exp \biggl( \sum_{j} \biggl[ \lambda_{s_{j}}(u_{j}, q_{j}) - i\epsilon p_{j}\frac {u_{j+1}-u_{j}}{\Delta t} \biggr] \frac{\Delta t}{\epsilon} \biggr) \\ &\quad\quad{}\times \exp \biggl(\bigl[i\epsilon p_{j}v_{n_{j}}(u_{j})-q_{j}v_{n_{j}}(u_{j}) \bigr]\frac{\Delta t}{\epsilon} \biggr). \end{aligned}$$

By inserting the eigenfunction products and using the Fourier representation of the Dirac delta function, we have introduced sums over the discrete labels $s_{j}$ and new phase variables $p_{j}$. However, this allows us to obtain a simple action principle in the limit $\epsilon\rightarrow0$. Since the path integral is ultimately independent of the $q_{j}$, we are free to set $q_{j}=i\epsilon p_{j}$ for all *j*, thus eliminating the final exponential factor. (Fixing the $q_{j}$ is analogous to gauge-fixing in field theory.) This choice means that we can perform the summations with respect to the intermediate discrete states $n_{j}$ using the orthogonality relation

$$ \sum_{n}R_{n}^{(s)}(u_{j},q_{j-1}) \xi_{n}^{(s')}(u_{j+1}, q_{j})=\delta _{s,s'} +O(\Delta u, \Delta q). $$

We thus obtain the result that $s_{j}=s$ for all *j*, which means that we can then take the continuum limit of (3.9) to obtain the following path integral from $u(0)=u_{0}$ to $u(\tau)=u$ (after performing the change of variables $i\epsilon p_{j}\rightarrow p_{j}$, that is, performing a contour deformation in the complex *p*-plane):

3.9 $$\begin{aligned} P(u,n,\tau|u_{0},n_{0},0) =&\sum _{s} \int_{u(0)=u_{0}}^{u(\tau)=u} \exp \biggl(-\frac{1}{\epsilon}{\int_{0}^{\tau}\bigl[p \dot{u}-\lambda _{s}(u,p)\bigr]\,dt} \biggr) \\ &{}\times R_{n}^{(s)}\bigl(u,p(\tau)\bigr) \xi_{n_{0}}^{(s)}\bigl(u_{0},p(0)\bigr) {\mathcal{D}}[p]{\mathcal{D}}[u]. \end{aligned}$$

Applying the Perron–Frobenius theorem to the linear operator on the left-hand side of (3.4) for fixed *u* and *q*, shows that there exists a real, simple Perron eigenvalue. We assume that the eigenvalues are ordered such that $\lambda_{0}> \operatorname{Re}(\lambda_{1})\geq \operatorname{Re}(\lambda_{2})\ldots$ with $\lambda_{0}$ the Perron eigenvalue. Since $\lambda_{0}$ is the only eigenvalue with a positive eigenfunction, we require on physical grounds that the initial and final states are only non-vanishing for $s=0$. It follows that the sum over *s* in (3.9) projects to the single term $s=0$. Also note that the factor $R_{n}^{(0)}(u,p(\tau))\xi _{n_{0}}^{(0)}(u_{0},p(0))$ in (3.9) essentially projects on to stochastic trajectories that start in the discrete state $n_{0}$ and terminate in the discrete state *n*. We will ignore any restrictions on these discrete states and simply consider the probability density (for fixed $u(0)=u_{0}$)

3.10 $$ P(u,t)=\int_{\mathbf{u}(0)=\mathbf{u}_{0}}^{\mathbf{u}(\tau )=\mathbf{u}} \mathrm {e}^{-S[u,p]/\epsilon} {\mathcal{D}}[p]{\mathcal{D}}[u], $$

with the action

3.11 $$ S[u,p]=\int_{0}^{\tau} \bigl[p\dot{u}- \lambda_{0}(u,p) \bigr]\,dt , $$

and $\lambda_{0}$ the Perron eigenvalue of the linear equation

3.12 $$\begin{aligned} \sum_{m} \bigl[W_{nm}(u) + p \delta_{n,m}v_{m}(u) \bigr]R_{m}^{(0)}(u, p) =\lambda_{0}(u, p)R_{n}^{(0)}(u, p). \end{aligned}$$

Formally comparing to classical mechanics, we have a path integral in a phase space $(u,p)$ consisting of a dynamical variable $u(t)$ and its ‘conjugate momentum’ $p(t)$ with the Perron eigenvalue $\lambda_{0}(u,p)$ interpreted as a Hamiltonian. This underlying Hamiltonian structure of a stochastic hybrid system has also been identified using large-deviation theory [34, 41]; see below.

### Large-Deviation Principles

It is important to point out that the formal derivation of the path integral (3.10), see also [33], involves a few steps that have not been justified rigorously. First, we ‘gauge fix’ the path integral by setting $q_{j}=\epsilon p_{j}$ with $p_{j}$ pure imaginary. However, when we carry out steepest descents, we assume that the dominant contribution to the path integral in the complex *p*-plane occurs for real $p_{j}$. (There is an assumption as regards analytic continuation.) This then allows us to apply the Perron–Frobenius theorem to the linear operator of the eigenvalue equation. Second, we have not established that the discrete path integral converges to a well-defined functional measure in the continuum limit. Nevertheless, it turns out that the resulting action $S[u,p]$ is identical to one obtained using large-deviation theory [41–43]. This connection has recently been established by Bressloff and Faugeras [34]. We briefly summarize the main results here.

Following [34], we take as our starting point a Lagrangian large-deviation principle of Faggionato et al. [42, 43], which applies to a wide class of stochastic hybrid systems. Here we state the LDP for the particular one-population neural model. Let $\mathcal {M}_{+}([0,T])$ denote the space of non-negative finite measures on the interval $[0,T]$ and take the *K*-dim. vector $\{\psi (t)\}_{t\in[0,T]}$ to be an element of the product space $\mathcal {M}_{+}([0,T])^{\varGamma}$ where $\varGamma=\{0,\ldots,{\mathcal{N}}\}$ and $K={\mathcal{N}}+1$. In other words, for each $t\in[0,T]$, $\psi (t)=(\psi_{1}(t),\ldots,\psi_{K}(t))$ such that

$$\psi_{n}(t)\geq0,\quad \sum_{n\in\varGamma} \psi_{n}(t)=1. $$

A particular realization of the stochastic process, $\{(x(t),n(t))\} _{t\in[0,T]}$, then lies in the product space $C([0,T]) \times \mathcal {M}_{+}([0,T])^{\varGamma}$ with

3.13 $$ \psi_{n}(t)=1_{\{n(t)=n\}}\equiv \begin{cases} 1, & \mbox{if } n(t)=n,\\ 0 , & \mbox{if } n(t)\neq n, \end{cases} $$

and

3.14 $$ u(t)=u_{0}+\int_{0}^{t} \sum_{n\in\varGamma} \psi_{n}(s)v_{n} \bigl(u(s)\bigr)\,ds. $$

Let ${\mathcal{Y}}_{u_{0}}$ denote the subspace of $C([0,T]) \times \mathcal {M}_{+}([0,T])^{\varGamma}$ for which (3.14) holds but *ψ* is now a general element of $\mathcal {M}_{+}([0,T])^{\varGamma}$. Such a space contains the set of trajectories of the stochastic hybrid system with $\psi_{n}(t)$ given by (3.13) and $n(t)$ evolving according to the Markov chain. Finally, take $P^{\epsilon}_{u_{0},n_{0}}$ to be the probability density functional or law of the set of trajectories in ${\mathcal{Y}}_{u_{0}}$. The following large-deviation principle then holds [42, 43]:

- *For any*$(u,\psi)\in C([0,T]) \times[0,1]^{\varGamma}$*define*
  3.15 $$ j(u,\psi)=\sup_{z \in(0,\infty)^{\varGamma}} \sum _{(n,n') \in\varGamma \times \varGamma} \psi_{n} W_{n'n}(u) \biggl[1- \frac{z_{n'}}{z_{n}} \biggr]. $$
  *Then, for any given path*$\{(u(t),\psi(t))\}_{t\in[0,T]} \in {\mathcal{Y}}_{u_{0}}$*,*
  3.16 $$ \mathbb {P}^{\epsilon}_{u_{0},n_{0}} \bigl[ \bigl\{ \bigl(u(t),\psi(t)\bigr)\bigr\} _{t \in[0,T]} \bigr]\sim \mathrm {e}^{-J_{T}(\{(u(t),\psi(t))\}_{t\in[0,T]})/ \epsilon}, $$
  *where the rate function*$J_{T}\,\colon {\mathcal{Y}}_{u_{0}} \to[0,\infty)$*is given by*
  3.17 $$ J_{T}\bigl(\bigl\{ \bigl(u(t),\psi(t)\bigr)\bigr\} _{t\in[0,T]}\bigr)=\int_{0}^{T} j\bigl(u(t), \psi(t)\bigr)\,dt. $$
  *Here the symbol* ∼ *means asymptotic logarithmic equivalence in the limit*$\epsilon\rightarrow0$*.*

A key idea behind the LDP is that a slow dynamical process coupled to the fast Markov chain on *Γ* rapidly samples the different discrete states of *Γ* according to some non-negative measure *ψ*. In the limit $\epsilon\rightarrow0$, one has $\psi\rightarrow \rho $, where *ρ* is the ergodic measure of the Markov chain. On the other hand, for small but non-zero *ϵ*, *ψ* is itself distributed according to the LDP (3.16), whereby one averages the different functions $v_{n}(x)$ over the measure *ψ* to determine the dynamics of the slow system. In our population model, we are interested in the synaptic current *u* (for a current-based or voltage-based model). Eliminating $\psi(t)$ using a contraction principle then leads to the following LDP for $\{u(t)\}_{t\in[0,T]}$ alone [41, 43]:

- *Given an element*$\{u(t)\}_{t\in[0,T]}\in C([0,T])$*, we have*
  $$\mathbb {P}^{\epsilon}_{u_{0},n_{0}} \bigl[ \bigl\{ u(t)\bigr\} _{t \in[0,T]} \bigr]\sim \mathrm {e}^{-J_{T}(\{u(t)\}_{t\in[0,T]})/\epsilon}, $$
  *where the rate function*$J_{[0,T]}\,\colon C([0,T],\varOmega) \to[0,\infty)$*is given by*
  3.18 $$ J_{T}\bigl(\bigl\{ u(t)\bigr\} _{t\in[0,T]}\bigr)= \inf_{\{\psi(t)\}_{t\in[0,T]}: \dot {u}(t)=\sum _{n}v_{n}(u)\psi_{n}} J_{T} \bigl(\bigl\{ \bigl(u(t),\psi(t)\bigr)\bigr\} _{t\in [0,T]}\bigr). $$

Roughly speaking, one can understand the contraction principle in terms of steepest descents. That is

$$\begin{aligned} \mathbb {P}^{\epsilon}_{x_{0},n_{0}} \bigl[\bigl\{ u(t)\bigr\} _{t \in[0,T]} \bigr] =&\int \mathbb {P}^{\epsilon}_{u_{0},j_{0}} \bigl[ \bigl\{ \bigl(u(t),\psi(t) \bigr)\bigr\} _{t \in[0,T]} \bigr] D[\psi] \\ \sim&\int \mathrm {e}^{-J_{T}(\{(u(t),\psi(t))\}_{t\in[0,T]})/\epsilon}D[\psi], \end{aligned}$$

where $D[\psi]$ is the appropriate functional measure on $\mathcal {M}_{+}([0,T])^{\varGamma}$. The path integral is then dominated by the infimum of the rate function in the limit $\epsilon\rightarrow0$.

In [34], it is proven that the rate function (3.18) can be written in the form of an action

3.19 $$ J_{T}\bigl(\bigl\{ u(t)\bigr\} _{t\in[0,T]}\bigr)= \int_{0}^{T}L(u,\dot{u})\,dt, $$

with Lagrangian given by

3.20 $$ L(u,\dot{u}) =\mu(u,\dot{u})\dot{u}-\lambda_{0}\bigl(u(t),\mu(u, \dot{u})\bigr), $$

where $\lambda_{0}(x,\mu)$ is the Perron eigenvalue of the linear equation

$$\sum_{m}W_{nm}(u)R_{m}^{(0)}+ \mu v_{n}(u)R_{n}^{(0)}=\lambda_{0} R_{n}^{(0)}, $$

and $\mu=\mu(u,\dot{u})$ the solution of the equation

3.21 $$ \dot{u}=\frac{\partial\lambda_{0}}{\partial\mu}\equiv\sum _{m}R_{m}^{(0)}(u,\mu) \xi_{m}^{(0)}(u,\mu)v_{m}(u) $$

with $\xi^{(0)}$ the adjoint eigenvector of $R^{(0)}$. Note that *μ* is a Lagrange multiplier which is introduced in order to impose the constraint $\dot{u}=\sum_{m}v_{n}(u)\psi_{n}$ when evaluating the infimum of (3.18). Given the Lagrangian *L*, we can determine a corresponding Hamiltonian *H* according to the Fenchel–Legendre transformation

3.22 $$ H(u,p)= \sup_{y} \bigl[\bigl(p-\mu(u,y)\bigr)y+\lambda\bigl(u,\mu (u,y) \bigr) \bigr]. $$

Minimizing the right-hand side yields the equation

3.23 $$ p-\mu(u,y)+ \biggl[\frac{\partial\lambda}{\partial\mu}-y \biggr] \frac {\partial\mu}{\partial y}=0. $$

Since $\partial_{\mu}\lambda=y$, we see that $p=\mu$ i.e., we can identify the Lagrange multiplier *μ* in the construction of the Lagrangian as the conjugate momentum *p* of the Hamiltonian

3.24 $$ H=\lambda_{0}(u,p), $$

where $\lambda_{0}(u,p)$ is the Perron eigenvalue of the linear equation (3.12). It follows that the action obtained from a large-deviation principle is identical to the action (3.11) derived using formal path-integral methods.

### Calculation of Perron Eigenvalue

In our previous work [32, 33], we obtained an explicit solution for the Perron eigenvalue and the associated positive eigenvector by taking the number of discrete states in each population to be infinite, that is, ${\mathcal{N}}\rightarrow\infty$. However, the classical Perron–Frobenius theorem applies to finite-dimensional Markov processes. One consequence of this is that the Perron eigenvalue develops singularities in the thermodynamic limit. In order to explore this issue, let us return to the one-population eigenvalue equation (3.12), which takes the explicit form

3.25 $$\begin{aligned} 0 =& F(u)R_{n-1}^{(0)}(u,p)-\bigl[\lambda_{0}+F(u)+pu+n(1-wp) \bigr]R_{n}^{(0)}(u,p) \\ &{} +(n+1)R_{n+1}^{(0)}(u,p). \end{aligned}$$

In the infinite-dimensional case, one can formally solve this equation using the trial positive solution

3.26 $$ R_{n}^{(0)}(u,p)=\frac{\varLambda(u,p)^{n}}{n!},\quad \varLambda(u)=\lambda _{0}+F(u)+pu. $$

This yields the following equation relating *Λ* and *p*:

$$ \biggl[\frac{ F(u)}{\varLambda}-1 \biggr]n+\varLambda-F(u)-\lambda_{0}=-p(-u+wn). $$

We now collect terms independent of *n* and linear in *n*, respectively, to obtain the pair of equations

$$ p=-\frac{1}{w} \biggl[\frac{F(u)}{\varLambda}-1 \biggr],\quad \varLambda= F(u)+p u+ \lambda_{0}. $$

It follows that

3.27 $$ \varLambda=\frac{F(u)}{1-wp}, \qquad\lambda_{0}=pw\frac{F(u)}{1-wp}-pu. $$

There is clearly a singularity at $p=1/w$ such that $\varLambda(u,p)<0$ for $p>1/w$, contradicting the requirement that the eigenfunction $R_{n}^{(0)}$ is positive.

The origin of the singularity can be understood by considering a large, but finite population size ${\mathcal{N}}$. The Perron–Frobenius theorem then holds but the solution of the eigenvalue equation becomes non-trivial. The basic difficulty arises because the above ansatz for $R_{n}^{(0)}$ does not satisfy the boundary condition at $n={\mathcal{N}}$. That is, setting $n={\mathcal{N}}-1$ and $n={\mathcal{N}}$ in (3.25) with $R_{{\mathcal{N}}+1}^{(0)}=0$ gives

$$F(u)R_{{\mathcal{N}}-2}^{(0)}(u,p)-\bigl[\varLambda(u)+({\mathcal{N}}-1) (1-wp)\bigr]R_{{\mathcal{N}}-1}^{(0)}(u,p) +{\mathcal{N}}R_{{\mathcal{N}}}^{(0)}(u,p) =0 $$

and

$$F(u)R_{{\mathcal{N}}-1}^{(0)}(u,p)-\bigl[\varLambda(u)-F(u)+{\mathcal{N}}(1-wp)\bigr]R_{\mathcal{N}}^{(0)}(u,p) =0. $$

Assuming that $R_{n}^{(0)}=\varLambda^{n}/n!$ for $0\leq n <{\mathcal{N}}$, with *Λ* given by (3.26) and $p<1/w$ (positive solution), we see that the first equation is satisfied by taking $R_{\mathcal{N}}^{(0)}=\varLambda^{\mathcal{N}}/{\mathcal{N}}!$. However, the second equation requires

$$R_{\mathcal{N}}^{(0)}=\frac{F(u)}{\varLambda(u)-F(u)+{\mathcal{N}}(1-wp)}R_{{\mathcal{N}}-1}^{(0)}(u). $$

In the large-*N* limit with $p<1/w$, we set

$$R_{\mathcal{N}}^{(0)}\rightarrow\frac{F(u)}{{\mathcal{N}}(1-wp)}R_{{\mathcal{N}}-1}^{(0)}(u)= \frac{\varLambda(u)}{{\mathcal{N}}}R^{(0)}_{{\mathcal{N}}-1}(u). $$

This shows that the given ansatz is a good approximation to the eigensolution for large ${\mathcal{N}}$ and $p<1/w$. Clearly, the given ansatz breaks down as *p* crosses $p=1/w$. Although the Perron–Frobenius theorem guarantees a unique positive solution for finite ${\mathcal{N}}$, it does not have a simple expression in the large *N* limit. In conclusion, our expression (3.27) for the Perron eigenvalue only holds for $p<1/w$. This does not affect our subsequent analysis because we evaluate the path integral in regions for which $p < 1/w$.

### Multi-population Model

Following along identical lines to the one-population model, we can derive a path-integral representation of the solution of the multi-population CK equation (2.6):

3.28 $$ p(\mathbf{u},\tau)=\int_{\mathbf{u}(0)=\mathbf{u}_{0}}^{\mathbf{u}(\tau)=\mathbf{u}} {\mathcal{D}}[{ \mathbf{p}} ]{\mathcal{D}}[\mathbf{u}] \exp \biggl(-\frac{1}{\epsilon}S[\mathbf{u},{ \mathbf{p}}] \biggr) $$

with the action

3.29 $$ S[\mathbf{u},{\mathbf{p}}]=\int_{0}^{\tau} \Biggl[\sum _{\alpha =1}^{M}p_{\alpha} \dot{u}_{\alpha }-\lambda_{0}(\mathbf{u},{\mathbf{p}}) \Biggr]\,dt . $$

Here $\lambda_{0}$ is the Perron eigenvalue of the following linear operator equation (cf. (3.4)):

3.30 $$\begin{aligned} &\sum_{\mathbf{m}}W(\mathbf{n},\mathbf{m};\mathbf{u}){R}^{(0)}(\mathbf{u},{\mathbf{p}},\mathbf{m}) \\ &\quad=\Biggl[\lambda_{0}( \mathbf{u},{\mathbf{p}})-\sum_{\alpha =1}^{M} p_{\alpha}v_{\alpha}(\mathbf{u},\mathbf{n}) \Biggr] {R}^{(0)}(\mathbf{u},{\mathbf{p}},\mathbf{n}), \end{aligned}$$

and $\xi^{(0)}$ is the corresponding adjoint eigenvector. For sufficiently small $p_{\alpha}$s, (3.30) can be solved for the Perron eigenvalue in the thermodynamic limit ${\mathcal{N}}\rightarrow \infty$ using the ansatz

3.31 $$ {R}^{(0)}(\mathbf{u},{\mathbf{p}},\mathbf{n})=\prod_{\alpha=1}^{M} \frac {\varLambda_{\alpha}(\mathbf{u},{\mathbf{p}} )^{n_{\alpha}}}{n_{\alpha}!}. $$

Substituting into (3.30) and using the explicit expressions for *W* and $v_{\alpha}$, we find that

3.32 $$\begin{aligned} &\sum_{\alpha=1}^{M} \biggl( \biggl[\frac{F(u_{\alpha})}{\varLambda _{\alpha }}-1 \biggr]n_{\alpha}+\varLambda_{\alpha} -F(u_{\alpha}) \biggr)-\lambda _{0} \\ &\quad =-\sum_{\alpha=1}^{M} p_{\alpha} \biggl[-u_{\alpha}+\sum_{\beta }w_{\alpha\beta}n_{\beta} \biggr]. \end{aligned}$$

Collecting terms in $n_{\alpha}$ for each *α* yields

3.33 $$ \frac{F(u_{\alpha})}{\varLambda_{\alpha}}-1 = -\sum_{\beta=1}^{M} p_{\beta }w_{\beta\alpha}, $$

and collecting terms independent of all $n_{\alpha}$ gives

3.34 $$ \lambda_{0}=\sum_{\alpha=1}^{M} \bigl[\varLambda_{\alpha}-F(u_{\alpha })-u_{\alpha}p_{\alpha} \bigr]. $$

Solving for each $\varLambda_{\alpha}$ in terms of **p**, we have

3.35 $$ \lambda_{0}(\mathbf{u},{\mathbf{p}}) \equiv\sum _{\alpha=1}^{M} \biggl[ \frac{F(u_{\alpha })}{1-\sum_{\beta=1}^{M} p_{\beta}w_{\beta\alpha}}-u_{\alpha }p_{\alpha }-F(u_{\alpha}) \biggr]. $$

As in the one-population model, the Perron eigenvalue has singularities, reflecting the possible breakdown of the Perron–Frobenius theorem in the thermodynamic limit.

## A Variational Principle and Optimal Paths of Escape

It is clear from the formal structure of the path integral (3.28) that each synaptic variable $u_{\alpha}$ has a ‘conjugate momentum’ $p_{\alpha}$ with $\lambda_{0}(\mathbf{u},{\mathbf{p}})$ the corresponding ‘Hamiltonian’ *H*. Applying steepest descents to the path integral for small *ϵ* yields a variational principle in which maximum-likelihood paths minimize the action (3.29). As is well known from classical mechanics, the least action principle leads to Hamilton’s equations

**Fig. 1:**
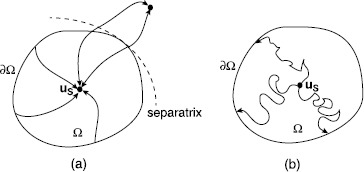
**a** Deterministic trajectories of a multistable dynamical system. The subset *Ω* is contained within the basin of attraction of a fixed point $\mathbf {u}_{s}$. The boundary of the basin of attraction consists of separatrices, which are also solution trajectories. Paths that start in a neighborhood of a separatrix are attracted by different fixed points, depending whether they begin on the left or right of the separatrix. **b** Random trajectories of the stochastic system. Escape from the domain *Ω* occurs when a random trajectory hits the boundary *∂Ω*

4.1 $$ \dot{\mathbf{u}} = \nabla_{{\mathbf{p}}}{H}(\mathbf{u}, {\mathbf{p}}), \quad\quad\dot{{\mathbf{p}}} = -\nabla_{\mathbf{u}}{H}(\mathbf{u} , {\mathbf{p}}), $$

describing a ‘classical particle’ moving in the phase space $(\mathbf{u},{\mathbf{p}})$. What is the physical interpretation of the solutions to Hamilton’s equations? In order to address this question, suppose that the underlying deterministic mean-field equation (2.19) has a stable fixed point $\mathbf{u}_{s}$ with some basin of attraction *Ω*, as illustrated in Fig. 1. If the system starts within *Ω*, then on relatively short time scales we expect the system to rapidly converge to $\mathbf{u}_{s}$ along a classical deterministic trajectory, with noise generating Gaussian-like fluctuations about this trajectory. However, on a longer time scale, a rare event (large fluctuation) will generate a path of escape from $\mathbf{u}_{s}$ to the boundary of *Ω*. It turns out that both classical trajectories and the maximum-likelihood paths of escape correspond to zero energy solutions of Hamilton’s equations of motion; this follows from the fact that the action vanishes at fixed points of the deterministic mean-field equation. We will illustrate this by considering the simpler one-population model.

Setting $\lambda_{0}=0$ in the eigenvalue equation (3.12) gives

4.2 $$ \sum \bigl[W_{nm}(u) + p \delta_{n,m}v_{m}(u) \bigr]R_{m}^{(0)}(u, p) =0 $$

with $R_{m}^{(0)}$ required to be a positive function. One solution is $p=0$ and $R_{m}^{(0)}(u, 0) =\rho_{m}(u)$ with

$$ \sum_{m}W_{nm}(u) \rho_{m}(u) =0. $$

Differentiating the eigenvalue equation with respect to *p* and then setting $p=0$, $\lambda_{0}=0$ shows that

$$\frac{\partial\lambda_{0}(u,p)}{\partial p} \bigg|_{p=0}\rho _{n}(u)=v_{n}(u) \rho_{n}(u)+\sum_{m}W_{nm}(u) \frac{\partial R_{m}^{(0)}(u,p)}{\partial p} \bigg|_{p=0}. $$

Summing both sides with respect to *n* and using $\sum_{n}W_{nm}=0$,

$$\frac{\partial\lambda_{0}(u,p)}{\partial p} \bigg|_{p=0}=\sum_{n} v_{n}(u)\rho_{n}(u). $$

Similarly, one finds that ${\partial\lambda_{0}(u,p)}/{\partial u}$ vanishes at $p=0$. Hence, Hamilton’s equations

$$\dot{u}=\frac{\partial\lambda_{0}(u,p)}{\partial p},\quad\quad \dot {p}=-\frac {\partial\lambda_{0}(u,p)}{\partial u} $$

reduce to

$$\dot{u}=\sum_{n} v_{n}(u) \rho_{n}(u),\quad\quad \dot{p} =0 \quad\mbox{for } p=0. $$

It follows that $(u_{s},0)$ is a fixed point in the full phase space with the line $p=0$ a stable manifold. Along this manifold, *u* converges to $u_{s}$ according to the scalar version of the mean-field equation (2.19). From the explicit expression for $\lambda_{0}(u,p)$, (3.27), we see that there exists another zero energy solution given by

$$p=\phi(u)\equiv\frac{1}{w}-\frac{F(u)}{u}, $$

which is the unique, non-trivial solution of the equation

4.3 $$ \sum_{m} \bigl[W_{nm}(u) + \phi(u) \delta_{n,m}v_{m}(u) \bigr]\psi _{m}(u) =0 $$

for positive functions $\psi_{m}(u)$. (Note that $p<1/w$ so that we do not have to worry about the singular nature of the Perron eigenvalue in the limit ${\mathcal{N}}\rightarrow\infty$.) It corresponds to the trajectory along the unstable manifold of $(u_{s},0)$ and is the optimal path of escape from $u_{s}$. Along this optimal path $\lambda_{0}=0$, so that the corresponding action is given by the quasipotential

4.4 $$ \varPhi(u)= \int_{-\infty}^{t}\bigl[p\dot{u}- \lambda_{0}(u,p)\bigr]\,dt \bigg|_{p=\phi(u)}=\int_{-\infty}^{t} \phi(u)\dot{u}\,dt=\int_{u_{s}}^{u}\phi(y)\,dy $$

and

4.5 $$ P\sim \mathrm {e}^{-\varPhi(u)/\epsilon}. $$

A similar situation holds for the higher-dimensional case, except that there are now multiple maximum-likelihood paths of escape from a metastable state [27, 33].

## The Diffusion Approximation and Neural Pattern Formation

Another useful application of the multi-population path integral (3.28) is that it provides a direct method for obtaining a Gaussian or diffusion approximation of the stochastic hybrid system, equivalent to the one obtained using the more complicated QSS reduction [27]. Performing the rescaling ${\mathbf{p}}\rightarrow i{\mathbf{p}}/\epsilon$ gives

$$\begin{aligned} P(\mathbf{u},t) =&\int_{\mathbf{u}(0)=\mathbf{u}_{0}}^{\mathbf{u}(\tau)=\mathbf{u}} D[\mathbf{u}]D[{\mathbf{p}}] \\ &{}\times \exp \biggl(-\int_{0}^{\tau}i\sum _{\alpha}p_{\alpha} \biggl[ \dot{u}_{\alpha }+u_{\alpha }- \sum_{\beta}\frac{w_{\alpha\beta}F(u_{\beta})}{1-i\epsilon\sum_{\gamma}w_{\gamma\beta}p_{\gamma}} \biggr]\,dt \biggr). \end{aligned}$$

The Gaussian approximation involves Taylor expanding the Lagrangian to first order in *ϵ*, which yields a quadratic in *p*:

$$\begin{aligned} P(u,t) =&\int_{\mathbf{u}(0)=\mathbf{u}_{0}}^{\mathbf{u}(\tau )=\mathbf{u}} D[\mathbf{u}]D[{\mathbf{p}}] \exp \biggl(\int_{0}^{\tau} i\sum _{\alpha}p_{\alpha} \biggl(\dot{u}_{\alpha }+u_{\alpha }- \sum_{\beta}w_{\alpha\beta}F(u_{\beta}) \biggr) \biggr) \\ &{} \times\exp \biggl( -i\epsilon\int_{0}^{\tau} \sum _{\alpha ,\gamma }p_{\alpha}{\mathcal{Q}}_{\alpha\gamma}( \mathbf{u})p_{\gamma}\,dt \biggr), \end{aligned}$$

where ${\mathcal{Q}}_{\alpha\gamma}(\mathbf{u})=\sum_{\beta}w_{\alpha \beta }F(u_{\beta})w_{\gamma\beta}$. Performing the Gaussian integration then yields

$$P(\mathbf{u},t)=\int D[\mathbf{u}]\mathrm {e}^{-{\mathcal{A}}[\mathbf{u}]/\epsilon}, $$

with action functional

5.1 $$ {\mathcal{A}}[\mathbf{u}]=\frac{1}{4}\int_{0}^{\tau} \sum_{\alpha ,\beta} \bigl(\dot{u}_{\alpha}(t)-V_{\alpha} \bigl(\mathbf{u}(t)\bigr) \bigr){\mathcal{Q}}^{-1}_{\alpha \beta}(\mathbf{u}) \bigl(\dot{u}_{\beta}(t)-V_{\beta}\bigl(\mathbf{u}(t)\bigr) \bigr)\,dt, $$

where $V_{\alpha}(\mathbf{u})=-u_{\alpha}+\sum_{\beta}w_{\alpha \beta}F(u_{\beta })$. This path integral is identical in form to the Onsager–Machlup path-integral representation [44] of solutions to the Langevin equation

5.2 $$ dU_{\alpha}(t)=V_{\alpha}(\mathbf{U})\,dt +\sqrt{2 \epsilon}\sum_{\beta }w_{\alpha\beta} \sqrt{F(u_{\beta})}\,dW_{\beta}(t), $$

where the $W_{\alpha}(t)$ are independent Wiener processes. Since there is no additional Jacobian factor in the Onsager–Machlup path integral, it follows that the Langevin equation is of the Ito form. As we have discussed extensively elsewhere [27, 33], the diffusion or Gaussian approximation breaks down when solving escape problems. On the other hand, it provides useful information when analyzing the effects of fluctuations within the basin of attraction of a metastable state. For example, it is well known within the context of PDEs that fluctuations can enlarge the parameter regime over which time-periodic (limit cycles) or spatially periodic (Turing patterns) can occur. A similar phenomenon exists for stochastic hybrid neural networks. We will illustrate this by considering Turing-like instabilities in a spatially structured hybrid neural network under the diffusion approximation.

### Noise-Induced Pattern Formation

Consider a system of coupled homogeneous neural populations that are distributed on a regular *d*-dimensional lattice ℒ, with lattice spacing Δ*α* and site index $\alpha\in {\mathcal{L}}$. Following recent studies of stochastic pattern formation in RD systems [45–51], we investigate the occurrence of stochastic neural patterns by linearizing the spatially discrete Langevin equation (5.2) about a homogeneous stationary solution $u_{0}$ of the mean-field equation (2.19) and calculating the resulting power spectrum using discrete Fourier transforms. In order to reflect the homogeneous structure of the weights we also set

$$w_{\alpha\alpha'}=w\bigl(\bigl|\alpha-\alpha'\bigr|\bigr). $$

Substituting

$$U_{\alpha}(t)=u_{0}+\sqrt{\epsilon}\varPhi_{\alpha}(t) $$

into (5.2) and Taylor expanding to first order in *Φ* gives the multivariate Ornstein–Uhlenbeck process

5.3 $$ \frac{d\varPhi_{\alpha}}{dt}=\sum_{\alpha'}J_{0} \bigl(\bigl|\alpha-\alpha '\bigr|\bigr)\varPhi _{\alpha'}(t)+\sum _{\alpha'}B_{0}\bigl(\bigl|\alpha- \alpha'\bigr|\bigr)\xi_{\alpha'}(t), $$

with

5.4 $$ J_{0}\bigl(\bigl|\alpha-\alpha'\bigr|\bigr)= \frac{\partial{V}_{\alpha}}{\partial U_{\alpha'}} \bigg|_{u_{0}}=-\delta_{\alpha,\alpha'}+w\bigl(\bigl|\alpha -\alpha'\bigr| \bigr)F'(u_{0}), $$

and

5.5 $$ B_{0}\bigl(\bigl|\alpha-\alpha'\bigr|\bigr)=w \bigl(\bigl|\alpha-\alpha'\bigr|\bigr)\sqrt{2F(u_{0})}. $$

Considerable insight into the behavior of the system can now be obtained by transforming to Fourier space [45, 51]. For simplicity, consider a 1D lattice with periodic boundary conditions, $u_{\alpha+N}=u_{\alpha}$ for $\alpha= 1,\ldots, N$ and set the lattice spacing $\Delta\alpha=1$. Introduce the discrete Fourier transforms

$$\widehat{\varPhi}(k)= \sum_{\alpha} \mathrm {e}^{-ik \alpha} \varPhi_{\alpha },\quad \varPhi _{\alpha}=\frac{1}{N}\sum _{k}\mathrm {e}^{ik \alpha}\widehat{\varPhi}(k) $$

with $k=2\pi m/N$, $m=0,\ldots, N-1$. Using the following result for convolutions:

$$\begin{aligned} \sum_{\alpha,\alpha'}\mathrm {e}^{-ik \alpha}J\bigl(\alpha- \alpha'\bigr)\varPhi _{\alpha'}= \widehat{J}(k)\widehat{ \varPhi}(k), \end{aligned}$$

the discrete Fourier transform of the Langevin equation is

5.6 $$ \frac{d\widehat{\varPhi}(k,t)}{dt}= \widehat{J}_{0}(k)\widehat{\varPhi }(k,t)+\widehat{B}_{0}(k)\widehat{\xi}(k,t) $$

with

5.7 $$ \widehat{J}_{0}(k)=-1+\widehat{w}(k)F'(u_{0}),\quad\quad \widehat {B}_{0}(k)=\widehat{w}(k)\sqrt{2F(u_{0})} $$

and $\langle\widehat{\xi}(k,t)\rangle=0$,

$$\bigl\langle \widehat{\xi}(k,t)\widehat{\xi}\bigl(k',t' \bigr)\bigr\rangle =\delta _{k,-k'}\delta\bigl(t-t'\bigr). $$

Note that the homogeneous equation

5.8 $$ \frac{d\widehat{\varPhi}(k,t)}{dt}= \widehat{J}_{0}(k)\widehat{ \varPhi}(k,t) $$

determines the stability of the fixed point $u_{0}$ in the absence of noise. It follows that the deterministic system is stable provided that $\widehat{J}_{0}(k) <0$ for all *k*. Suppose that the gain $\mu=F'(u_{0})$ is treated as a bifurcation parameter. Clearly if $\widehat{w}(k)$ is bounded and *μ* is sufficiently small, then $\widetilde{J}_{0}(k) <0$ for all *k*. However, if $\max_{k}\{\widehat{w}(k)\}=\widehat{w}(k_{c}) >0$ then the fixed point becomes marginally stable at $\mu=\mu_{c}=1/ \widehat{w}(k_{c}) $, resulting in the growth of a spatially periodic pattern of wavenumber $k_{c}$ as *μ* crosses $\mu_{c}$. A standard neural mechanism for inducing a Turing-like instability is to have a combination of short-range excitation and longer-range inhibition [52, 53]. This can implemented in the 1D scalar model by taking *w* to be the difference-of-Gaussians

5.9 $$ w_{D}(x) = \mathrm{e}^{-x^{2} /2} - A \mathrm{e}^{-x^{2} /2\sigma^{2}}. $$

(More precisely, in order to match the periodic boundary conditions, we should take $w(\alpha)=\sum_{n}w_{D}(\alpha-nN)$.)

Spectral theory can now be used to determine the effects of noise on pattern formation. First, Fourier transforming the Langevin equation (5.6) with respect to time gives

$$\varLambda(k,\varOmega)\widehat{\varPhi}(k,\varOmega)=\widehat {B}_{0}(k)\widehat{\xi }(k,\varOmega) $$

with

$$\varLambda(k,\varOmega)=-i\varOmega-\widehat{J}_{0}(k) $$

and

$$\bigl\langle \widehat{\xi}(k,\varOmega)\bigr\rangle =0, \quad\quad\bigl\langle \widehat {\xi }(k,\varOmega) \widehat{\xi}\bigl(k',\varOmega'\bigr) \bigr\rangle =\delta_{k,-k'}\delta \bigl(\varOmega+\varOmega' \bigr). $$

It follows that

$$\begin{aligned} \bigl\langle \widehat{\varPhi}(k,\varOmega)\widehat{\varPhi} \bigl(k',\varOmega '\bigr)\bigr\rangle =& \biggl\langle \biggl[\frac{\widehat{B}_{0}(k)}{\varLambda(k,\varOmega )}\widehat {\xi}(k,\varOmega) \biggr] \biggl[ \frac{\widehat{B}_{0}(k')}{\varLambda (k',\varOmega ')}\widehat{\xi}\bigl(k',\varOmega' \bigr) \biggr] \biggr\rangle \\ =&\delta_{k,-k'}\delta\bigl(\varOmega+\varOmega'\bigr) \frac{|B_{0}(k)|^{2}}{|\varLambda (k,\varOmega)|^{2}}. \end{aligned}$$

Defining the power spectrum by

$$\bigl\langle \widehat{\varPhi}(k,\varOmega) \widehat{\varPhi} \bigl(k',\varOmega '\bigr)\bigr\rangle =S(k,\varOmega) \delta_{k,-k'}\delta\bigl(\varOmega+\varOmega'\bigr), $$

we deduce that

5.10 $$ S(k,\varOmega)= \frac{|B_{0}(k)|^{2}}{|\varLambda(k,\varOmega)|^{2}}. $$

From the deterministic theory, we know that the system undergoes a Turing instability (stationary patterns) rather than a Turing–Hopf instability (oscillatory patterns) so we can set $\varOmega=0$ and determine conditions under which $S(k,0)$ has a peak at a non-zero, finite value of *k*, which is an indication of a stochastic pattern. Substituting the explicit expression for $\varLambda(k,0)$ and $B_{0}(k)$, we have

5.11 $$ S(k,0)=\frac{2\widehat{w}(k)^{2}F(u_{0})}{[-1+\widehat {w}(k)F'(u_{0})]^{2}}=\frac{2F(u_{0})}{F'(u_{0})^{2}}\bigl[1+\lambda(k)^{-1} \bigr]^{2}. $$

Suppose that $\mu\equiv F'(u_{0})<\mu_{c}$ so the system is below the deterministic critical point for a Turing instability. Clearly $S(k,0)$ becomes singular as $\mu\rightarrow\mu_{c}$, consistent with the fixed point becoming unstable. The main new result is that $S(k,0)$ has a peak at the critical wavenumber $k_{c}$ for all *μ*, $0<\mu< \mu _{c}=\widehat{w}(k_{c})^{-1}$. This follows from the fact that $\lambda (k)<0$ for all *k* in the subcritical regime with $\min_{k}\{ |\lambda(k)|\} =|\lambda(k_{c})|$. Hence, $S(k,0)$ will have a peak at $k=k_{c}$ provided that

5.12 $$ 0< \bigl|\lambda(k_{c})\bigr|\equiv1- \mu\widehat{w}(k_{c})<1 \quad\implies\quad\mu<\mu_{c}. $$

**Fig. 2:**
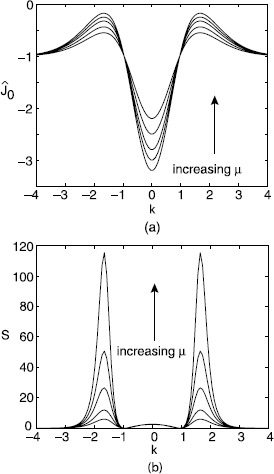
*Stochastic pattern formation in a scalar neural network*. **a** Plot of Fourier transformed weight distribution as a function of wavenumber *k* for various values of gain $\mu=f'(u_{0})$: $\mu=0.12,0.15,0.18,0.2,0.22$. **b** Sketch of corresponding power spectra $S(k,0)$, showing the peak in the spectrum at the critical wavenumber $k_{c}$ for $\mu <\mu_{c}$. Parameter values are $\sigma^{2}=4$, $A=0.5$, $F_{0}=20$, $\eta=2$, $\kappa=0$

This is illustrated in Fig. 2.

### Continuum Limit

The above stochastic model of a spatially structured lattice of neural populations can be reduced to a stochastic neural field by taking a continuum limit. A heuristic derivation proceeds as follows. Suppose that there is a uniform density *ρ* of populations distributed in ${\mathbb{R}}^{d}$. We then reinterpret $u_{\alpha}$ as the mean current averaged over the $\rho\Delta\alpha^{d}$ populations in the infinitesimal volume $\Delta\alpha^{d}$ centered at the lattice point $\alpha\in {\mathcal{L}}$. If an individual population in the set of populations centered at *α* is labeled by the pair $(\alpha,j)$, then

$$u_{\alpha}=\frac{\sum_{j} u_{\alpha,j}}{\rho\Delta\alpha^{d}}. $$

We will assume that the weights are slowly varying on the length-scale Δ*α* so that $w_{\alpha j,\alpha'j'}=w_{\alpha\alpha'}$. (Relaxing this assumption can lead to additional sources of stochasticity as explored in [12, 14].) The deterministic mean-field (2.19) for an individual population becomes

$$\tau\frac{du_{\alpha j}}{dt}= -{u_{\alpha j}(t)}+\sum _{\alpha '}w_{{\alpha}\alpha'}\sum_{j'} F(u_{\alpha'j'}). $$

Averaging with respect to *j* gives

$$\begin{aligned} \tau\frac{du_{\alpha}}{dt} =& -{u_{\alpha}(t)}+\rho\Delta\alpha ^{d} \sum_{\alpha'}w_{{\alpha}\alpha'}\frac{1}{\rho\Delta\alpha ^{d}}\sum _{j'} F(u_{\alpha'j'}) \\ =&-{u_{\alpha}(t)}+\rho\Delta\alpha^{d}\sum _{\alpha'}w_{{\alpha }\alpha '}F(u_{\alpha}) \end{aligned}$$

under the approximation that all local populations are in a similar state so

$$\frac{1}{\rho\Delta\alpha^{d}}\sum_{j'} F(u_{\alpha',j'}) \approx F \biggl(\frac{1}{\rho\Delta\alpha^{d}}\sum_{j'}u_{\alpha',j'} \biggr). $$

Effectively, we are scaling the population firing-rate function by a factor $\rho\Delta\alpha^{d}$. Finally, setting $\alpha= \mathbf {x}$, $u_{\alpha}(t)=u(\mathbf {x},t)$, $\rho w_{\alpha\alpha'}=w(\mathbf {x},\mathbf {x}')$, and taking the continuum limit $\Delta \alpha\rightarrow0$ yields the deterministic neural field equation

5.13 $$ \tau\frac{\partial u}{\partial t}= -{u(\mathbf {x},t)}+\int_{{\mathbb{R}}^{d}}w\bigl(\mathbf {x},\mathbf {x}'\bigr)F\bigl(u\bigl(\mathbf {x}',t\bigr)\bigr)\,d \mathbf {x}'. $$

Applying a similar analysis to the diffusion matrix, we have

$$Q_{\alpha j,\alpha'j'}=\sum_{\alpha_{1},j_{1}}w_{\alpha\alpha _{1}}w_{\alpha '\alpha_{2}}F(u_{\alpha_{1},j_{1}}) =\rho\Delta\alpha^{d} \sum_{\alpha_{1}}w_{\alpha\alpha_{1}}w_{\alpha '\alpha _{2}}F(u_{\alpha_{1}}). $$

Hence, **Q** is independent of the local population labels *j*, $j'$ and the Langevin equation (5.2) becomes

5.14 $$ \frac{dU_{\alpha j}}{dt}=\overline{V}_{\alpha j}({\mathbf{U}})+\sqrt {{2 \epsilon \rho\Delta\alpha^{d}}}\sum_{\alpha'}B_{\alpha\alpha'}( {\mathbf{U}})\xi _{\alpha'}(t). $$

Averaging with respect to *j* and taking the continuum limit yields the following neural field model with spatiotemporal Gaussian white noise:

5.15 $$\begin{aligned} \frac{dU(\mathbf {x},t)}{dt} =& -U(\mathbf {x},t)+\int_{{\mathbb{R}}^{d}} w\bigl(\mathbf {x}, \mathbf {x}'\bigr)F\bigl(U\bigl(\mathbf {x}',t\bigr)\bigr)\,d \mathbf {x}' \\ &{} +\sqrt{\epsilon}\int_{{\mathbb{R}}^{d}}B\bigl(\mathbf {x},\mathbf {x}' \bigr)\xi\bigl(\mathbf {x}',t\bigr)\,d\mathbf {x}', \end{aligned}$$

where

5.16 $$ B\bigl(\mathbf {x},\mathbf {x}'\bigr)=w\bigl(\mathbf {x},\mathbf {x}'\bigr)\sqrt{2F \bigl(U\bigl(\mathbf {x}',t\bigr)\bigr)} $$

and $\langle\eta(\mathbf {x},t)\rangle= 0$,

5.17 $$ \bigl\langle \eta(\mathbf {x},t)\eta\bigl(\mathbf {x}',t'\bigr)\bigr\rangle =\delta\bigl(\mathbf {x}-\mathbf {x}'\bigr)\delta\bigl(t-t' \bigr). $$

For finite Δ*α*, we have introduced the scaling $\eta _{\alpha }(t)/\sqrt{\Delta\alpha^{d}}=\eta(\mathbf {x},t)$.

From a numerical perspective, any computer simulation would involve rediscretizing space and then solving a time-discretized version of the resulting stochastic neural field equation. On the other hand, in order to investigate analytically the effects of noise on spatiotemporal dynamics such as traveling waves, it is more useful to work directly with stochastic neural fields. One can then adapt various PDE methods for studying noise in spatially extended systems [15, 54–58]. Finally, note that a large-deviation principle for a stochastic neural field with additive noise has been developed in [59].

## Generating Functionals and the $1/\epsilon$ Loop Expansion

One step beyond the Gaussian approximation is to consider corrections to the mean-field equation (2.19), which couple the mean synaptic current with higher-order moments. As demonstrated previously for neural master equations [17, 18, 20], path integrals provide a systematic method for generating the hierarchy of moment equations. We will illustrate this by calculating the lowest-order correction to mean-field theory based on coupling to second-order correlations. One could then take investigate the bifurcation structure of the higher-dimensional dynamical system along analogous lines to Touboul and Ermentrout [13]. However, certain caution must be exercised, since one does not keep track of the validity of the truncated moment equations. Note that the path-integral methods used in this section were originally introduced within the context of stochastic processes by Martin–Siggia–Rose [60], and have previously been applied to stochastic neural networks by Sompolinsky et al. [61, 62] and Buice et al. [17, 20].

### Generating Functional and Moments

First note that the average synaptic current $U_{\alpha}$ is given by

6.1 $$\begin{aligned} \bigl\langle \!\bigl\langle U_{\alpha}(t_{1}) \bigr\rangle \!\bigr\rangle &=\int u_{\alpha }(t_{1})p[\mathbf{u},t_{1}]\,d \mathbf{u}=\int D[\mathbf{u}]D[{\mathbf{p}}] u_{\alpha}(t_{1}) \mathrm {e}^{-S[\mathbf{u},{\mathbf{p}} ]/\epsilon} \end{aligned}$$

and two-point correlations are

6.2 $$ \bigl\langle \!\bigl\langle U_{\alpha}(t_{1})U_{\beta}(t_{2}) \bigr\rangle \!\bigr\rangle =\int D[\mathbf{u} ]D[{\mathbf{p}}] u_{\alpha}(t_{1})u_{\beta}(t_{2}) \mathrm {e}^{-S[\mathbf{u},{\mathbf{p}}]/\epsilon}. $$

Another important characterization of the system is how the mean synaptic current responds to small external inputs. Suppose that we add a small external source term $h_{\alpha}(t)$ onto the right-hand side of the deterministic rate equation (2.19). Linearizing about the time-dependent solution of the unperturbed equation ($\mathbf{h}\equiv 0$) leads to the following (non-autonomous) linear equation for the perturbed solution $u_{\alpha}(t)=u_{\alpha}^{h}(t)-u_{\alpha}^{0}(t)$:

6.3 $$ \tau\frac{d u_{\alpha}}{dt}= -u_{\alpha}+\sum_{\beta}w_{\alpha \beta }F' \bigl(u_{\beta}^{0}\bigr)u_{\beta} +h_{\alpha}(t). $$

Introducing the Green’s function or propagator $G^{0}_{\alpha\beta }(t,t')$ according to the adjoint equation

6.4 $$\begin{aligned} -\tau\frac{d G^{0}_{\alpha\gamma}(t,t')}{dt'} =&-G^{0}_{\alpha\gamma }\bigl(t,t' \bigr)+ \sum_{\beta}w_{\alpha\beta}F' \bigl(u_{\beta}^{0}\bigr)G^{0}_{\beta \gamma } \bigl(t,t'\bigr) \\ &{}+\delta_{\alpha,\gamma}\delta\bigl(t-t' \bigr), \end{aligned}$$

we can express the linear response as

6.5 $$ u_{\alpha}(t)=\int^{t}\sum _{\gamma} G^{0}_{\alpha\gamma}\bigl(t,t' \bigr) h_{\gamma }\bigl(t'\bigr)\,dt' . $$

In other words, in terms of functional derivatives

6.6 $$ \frac{\delta u_{\alpha}(t)}{\delta h_{\gamma}(t')} = G^{0}_{\alpha \gamma}\bigl(t,t' \bigr). $$

Now suppose that we add a source term to the path-integral representation. This corresponds to adding a term $\int\sum_{\gamma} h_{\gamma}(t)p_{\gamma}(t)\,dt$ to the action (3.29). It follows that the associated Green’s function for the full stochastic model is given by

6.7 $$ {\mathcal{G}}_{\alpha\gamma}\bigl(t,t'\bigr)\equiv \frac{\delta\langle\!\langle U_{\alpha}(t)\rangle\!\rangle}{ \delta h_{\gamma}(t')} = \bigl\langle \!\bigl\langle U_{\alpha}(t)P_{\gamma} \bigl(t'\bigr)\bigr\rangle \!\bigr\rangle . $$

The above analysis motivates the introduction of the generating functional

6.8 $$\begin{aligned} Z[\mathbf{J},\widetilde{\mathbf{J}}] =&\int D[\mathbf{u}]D[{\mathbf{p}}] \\ &{}\times\exp \biggl(-\frac{S[\mathbf{u},{\mathbf{p}}]}{\epsilon}+\int dt \sum_{\alpha} \bigl[u_{\alpha}(t)\widetilde{J}_{\alpha}(t)+J_{\alpha }(t)p_{\alpha}(t) \bigr] \biggr). \end{aligned}$$

Various moments of physical interest can then be obtained by taking functional derivatives with respect to the ‘current sources’ **J**, $\widetilde{\mathbf{J}}$. For example,

$$\begin{aligned} \bigl\langle \!\bigl\langle U_{\alpha}(t)\bigr\rangle \!\bigr\rangle =& \frac{\delta }{\delta\widetilde{J}_{\alpha}(t)}Z[\mathbf{J},\widetilde{\mathbf{J}}] \bigg|_{\mathbf{J}=\widetilde{\mathbf{J}}=0}, \\ \bigl\langle \!\bigl\langle U_{\alpha}(t) U_{\beta} \bigl(t'\bigr)\bigr\rangle \!\bigr\rangle =& \frac{\delta}{\delta\widetilde{J}_{\alpha}(t)} \frac{\delta }{\delta \widetilde{J}_{\beta}(t)}Z[\mathbf{J},\widetilde{\mathbf{J}}] \bigg|_{\mathbf{J}= \widetilde{\mathbf{J}}=0}, \\ \bigl\langle \!\bigl\langle U_{\alpha}(t)P_{\beta} \bigl(t'\bigr)\bigr\rangle \!\bigr\rangle =& \frac{\delta}{\delta\widetilde{J}_{\alpha}(t)} \frac{\delta }{\delta J_{\beta}(t)}Z[\mathbf{J},\widetilde{\mathbf{J}}] \bigg|_{\mathbf{J}= \widetilde {\mathbf{J}}=0}. \end{aligned}$$

### Effective Action and Corrections to Mean-Field Equations

Let us rescale the currents according to $\mathbf{J} \rightarrow \mathbf{J}/\epsilon$ and $\widetilde{\mathbf{J}}\rightarrow \widetilde {\mathbf{J}}/\epsilon$ so that we can apply a loop expansion of the path integral (6.8), which is a diagrammatic method for carrying out an *ϵ* expansion based on steepest descents or the saddle-point method. First, we introduce the exact means

$$\nu_{\alpha}= \langle\!\langle U_{\alpha}\rangle \!\rangle,\quad\quad \widetilde {\nu}_{k}= \langle\!\langle P_{\alpha}\rangle\! \rangle, $$

and we shift the variables by

$$u_{\alpha}(t)\rightarrow u_{\alpha}(t)+\nu_{\alpha}(t),\quad\quad p_{\alpha }(t)= p_{\alpha}(t)+ \widetilde{\nu}_{\alpha}(t). $$

Expanding the action in (6.8) to second order in the shifted variables **u**, **p** yields an infinite-dimensional Gaussian integral, which can be formally evaluated to give

$$\begin{aligned} Z[\mathbf{J},\widetilde{\mathbf{J}}] \approx&\operatorname{Det} \bigl[{\mathcal{D}}[\boldsymbol{\nu},\widetilde{\boldsymbol{\nu} }] \bigr]^{-1/2} \\ &{} \times\exp \biggl(-\frac{S[\boldsymbol{\nu},\widetilde{\boldsymbol{\nu} }]}{\epsilon }+\frac{1}{\epsilon}\int dt \sum _{\alpha} \bigl[ \nu_{\alpha }(t)\widetilde{J}_{\alpha}(t)+{J}_{\alpha}(t) \widetilde{\nu }_{\alpha }(t) \bigr] \biggr), \end{aligned}$$

where ${\mathcal{D}}[\boldsymbol{\nu},\widetilde{\boldsymbol{\nu}}]$ is the matrix with components

6.9 $$ {\mathcal{D}}[\boldsymbol{\nu},\widetilde{\boldsymbol{\nu}}]_{r\alpha,s\beta } \bigl(t,t'\bigr)= \frac{\delta^{2} S}{\delta\mathbf{u}_{\alpha }^{r}(t)\delta\mathbf{u} _{\beta}^{s}(t')} \bigg|_{\mathbf{u}=\boldsymbol{\nu},{\mathbf{p}}=\widetilde{\boldsymbol{\nu}}}. $$

We have introduced the vectors $\mathbf{u}^{r}$, $r=1,2$ with $\mathbf{u}^{1}=\mathbf{u}$, $\mathbf{u}^{2}={\mathbf{p}}$. Using the following identity for a matrix **M**:

$$\operatorname{Det}\mathbf{M} =\mathrm {e}^{\operatorname{Tr}\log\mathbf{M}}, $$

we obtain the ${\mathcal{O}}(\epsilon)$ approximation

6.10 $$ Z[\mathbf{J},\widetilde{\mathbf{J}}]\approx \mathrm {e}^{-S_{\mathrm{eff}}[\boldsymbol{\nu },\widetilde{\boldsymbol{\nu}}]/\epsilon}\mathrm{e}^{\int dt \sum_{\alpha} [ \nu _{\alpha}(t)\widetilde{J}_{\alpha}(t)+{J}_{\alpha}(t)\widetilde {\nu }_{\alpha}(t) ]/\epsilon}, $$

where

6.11 $$ S_{\mathrm{eff}}[\boldsymbol{\nu},\widetilde{\boldsymbol{\nu}}]=S[\boldsymbol{ \nu},\widetilde{\boldsymbol{\nu}}]+\frac{\epsilon}{2} \operatorname{Tr}\log \bigl[{\mathcal{D}}[\boldsymbol{ \nu }, \widetilde{\boldsymbol{\nu}}] \bigr]. $$

In order to use the above expansion to determine corrections to the mean-field equations, it is first necessary to introduce a little more formalism. First, consider the Legendre transformation

6.12 $$ \varGamma[ \boldsymbol{\nu},\widetilde{\boldsymbol{\nu}}]= W[\mathbf{J}, \widetilde{\mathbf{J}}]+\int dt \sum_{\alpha} \bigl[{ \nu}_{\alpha}(t)\widetilde {J}_{\alpha }(t)+{J}_{\alpha}(t) \widetilde{\nu}_{\alpha}(t) \bigr], $$

where $W[\mathbf{J},\widetilde{\mathbf{J}}]= -N^{-1}\log Z[\mathbf{J},\widetilde {\mathbf{J}}]$ and *Γ* is known as the effective action. Since

6.13 $$ \nu_{\alpha}(t)= -\frac{\delta W}{\delta\widetilde {J}_{\alpha}(t)},\quad\quad \widetilde{\nu}_{\alpha}(t)= - \frac{\delta W}{\delta{J}_{\alpha}(t)}, $$

it follows from functionally differentiating (6.12) that

6.14 $$ \widetilde{J}_{\alpha}(t)= \frac{\delta\varGamma}{\delta{\nu }_{\alpha }(t)},\quad\quad {J}_{\alpha}(t)= \frac{\delta\varGamma}{\delta\widetilde {\nu }_{\alpha}(t)}. $$

Dynamical equations for the physical mean fields $\nu_{\alpha}(t)$ are then generated by setting $\mathbf{J}=0=\widetilde{\mathbf{J}}$ in (6.14). Another useful result is obtained by functionally differentiating (6.13) with respect to the mean fields ***ν***, $\widetilde{\boldsymbol{\nu}}$:

$$\begin{aligned} \delta\bigl(t-t'\bigr)\delta_{r,s}\delta_{\alpha,\beta} =& \frac{\delta\nu _{\alpha }^{r}(t)}{\delta\nu_{\beta}^{s}(t')} =-\frac{\delta^{2} W}{\delta J_{\alpha }^{r}(t)\delta\nu_{\beta}^{s}(t')} \\ =& -\sum_{q=1,2}\sum_{\gamma=1}^{M} \int\frac{\delta^{2} W}{\delta J_{\alpha}^{r}(t)\delta J_{\gamma}^{q}(\tau)}\frac{\delta J_{\gamma }^{q}(\tau )}{\delta\nu_{\beta}^{s}(t')}\,d\tau, \end{aligned}$$

where $\nu_{\alpha}^{1}=\nu_{\alpha}$, $\nu_{\alpha}^{2}=\widetilde{\nu }_{\alpha}$ and $J_{\alpha}^{1}=\widetilde{J}_{\alpha}$, $J_{\alpha }^{2}=J_{\alpha}$. Differentiating (6.14) with respect to **J**, $\widetilde{\mathbf{J}}$ then shows that

$$ \sum_{q=1,2}\sum_{\gamma=1}^{M} \int\frac{\delta^{2} W}{\delta J_{\alpha }^{r}(t)\delta J_{\gamma}^{q}(\tau)}\frac{\delta^{2} \varGamma}{\delta\nu _{\gamma}^{q}(\tau)\delta\nu_{\beta}^{s}(t')}\,d\tau=-\delta _{r,s}\delta _{k,l}\delta\bigl(t-t'\bigr). $$

In other words, defining the infinite-dimensional matrix $\widehat {\mathcal{D}} [\boldsymbol{\nu},\widetilde{\boldsymbol{\nu}}]$ according to

6.15 $$ \widehat{\mathcal{D}} [\boldsymbol{\nu},\widetilde{\boldsymbol{ \nu}}]_{r,s}\bigl(\alpha ,t;\beta,t'\bigr)= \frac{\delta^{2} \varGamma}{\delta\nu_{\alpha }^{r}(t)\delta\nu _{\beta}^{s}(t')}, $$

we see that $\widehat{\mathcal{D}}[\boldsymbol{\nu},\widetilde{\boldsymbol{\nu}}]$ is the inverse of the two-point covariance matrix with components

$$ C_{r\alpha,s\beta}\bigl(t,t'\bigr)\equiv- \frac{\delta^{2} W}{\delta J_{\alpha }^{r}(t)\delta J_{\beta}^{s}(t')}= \bigl[\bigl\langle \!\bigl\langle U_{\alpha}^{r}(t) U_{\beta}^{s}\bigl(t'\bigr)\bigr\rangle \!\bigr\rangle -\bigl\langle \!\bigl\langle U_{\alpha}^{r}(t) \bigr\rangle \! \bigr\rangle \bigl\langle \!\bigl\langle U_{\beta}^{s} \bigl(t'\bigr)\bigr\rangle \!\bigr\rangle \bigr]. $$

It now follows from (6.10) and (6.12) that $\varGamma[\boldsymbol{\nu},\widetilde{\boldsymbol{\nu}}]= S_{\mathrm{eff}}[(\boldsymbol{\nu },\widetilde{\boldsymbol{\nu}})] +{\mathcal{O}}(\epsilon^{2})$. Moreover, (6.9) and (6.15) imply that $\widehat{\mathcal{D}}[\boldsymbol{\nu },\widetilde{\boldsymbol{\nu}}] = {\mathcal{D}}[\boldsymbol{\nu},\widetilde{\boldsymbol{\nu} }]+{\mathcal{O}}(\epsilon)$, that is, we can take ${\mathcal{D}}[\boldsymbol{ \nu },\widetilde{\boldsymbol{\nu}}]$ to be the inverse of the two-point covariance matrix. The first-order correction to the mean-field equation (2.19) is then obtained from (6.14) after setting $\mathbf{J}=\widetilde{\mathbf{J}}=\widetilde{\boldsymbol{\nu}}=0$:

$$0= \frac{\delta\varGamma[\boldsymbol{\nu},\widetilde{\boldsymbol{\nu} }]}{\delta{\nu }_{\alpha}(t)} \bigg|_{\widetilde{\boldsymbol{\nu}}=0} = \frac {\delta S[\mathbf{u},{\mathbf{p}}]}{\delta p_{\alpha}(t)} \bigg|_{\mathbf{u}=\boldsymbol{\nu},{\mathbf{p}}=0} +\frac{\epsilon}{2}\operatorname{Tr} {\mathcal{D}}[\mathbf{u},{\mathbf{p}}]^{-1}\frac{\delta {\mathcal{D}}[\mathbf{u},{\mathbf{p}}]}{\delta p_{\alpha}(t)} \bigg|_{\mathbf{u}=\boldsymbol{\nu},{\mathbf{p}} =0}, $$

with

$$\begin{aligned} & \operatorname{Tr} {\mathcal{D}}[\mathbf{u},{\mathbf{p}}]^{-1} \frac{\delta {\mathcal{D}}[\mathbf{u},{\mathbf{p}} ]}{\delta p_{\alpha}(t)} \bigg|_{\mathbf{u}=\boldsymbol{\nu},{\mathbf{p}}=0} \\ &\quad=\int dt' \int dt'' \sum _{r\beta,s\gamma} C_{r\beta,s\gamma }\bigl(t',t'' \bigr) \frac{\delta}{\delta p_{\alpha}(t)} \frac{\delta^{2} S[\mathbf{u} ,{\mathbf{p}}]}{\delta u_{\beta}^{r}(t')u_{\gamma}^{s}(t'')} \bigg|_{\mathbf{u}=\boldsymbol{\nu},{\mathbf{p}}=0}. \end{aligned}$$

The functional derivative in the above equation forces $t=t'=t''$ (see also [20]). Since the only non-vanishing, equal-time two-point correlation function when ${\mathbf{p}}=0$ is for $r=s=1$, it follows that

$$ \operatorname{Tr} {\mathcal{D}}[\mathbf{u},{\mathbf{p}}]^{-1} \frac{\delta {\mathcal{D}}[\mathbf{u},{\mathbf{p}} ]}{\delta p_{\alpha}(t)} \bigg|_{\mathbf{u}=\boldsymbol{\nu},{\mathbf{p}}=0}=\sum_{\beta,\gamma } C_{\beta\gamma}(t)S_{\gamma\beta}^{\alpha}(t), $$

where

$$C_{\beta\gamma}(t)= \bigl[ \bigl\langle \!\bigl\langle U_{\beta}(t) U_{\gamma }(t)\bigr\rangle \!\bigr\rangle -\bigl\langle \!\bigl\langle U_{\beta}(t) \bigr\rangle \!\bigr\rangle \bigl\langle \! \bigl\langle U_{\gamma}(t)\bigr\rangle \!\bigr\rangle \bigr], $$

and

$$\frac{\delta}{\delta p_{\alpha}(t)} \frac{\delta^{2} S[\mathbf{u},{\mathbf{p}} ]}{\delta u_{\beta}(t')u_{\gamma}(t'')}\bigg|_{\mathbf{ u}=\boldsymbol{\nu },{\mathbf{p}} =0}=S_{\gamma\beta}^{\alpha}(t) \delta\bigl(t-t'\bigr)\delta\bigl(t-t'' \bigr). $$

Evaluating the functional derivative of the action *S* given by (3.29) and (3.35) finally yields the lowest-order correction to the mean-field equation (2.19), which could not be obtained from the Langevin equation (5.2):

6.16 $$ \tau\frac{du_{\alpha}}{dt}= -{u_{\alpha}(t)}+\sum _{\beta=1}^{M} w_{{\alpha}\beta}F(u_{\beta})- \frac{\epsilon}{2}\sum_{\beta }w_{\alpha \beta}C_{\beta\beta}(t) F''\bigl(u_{\beta}(t)\bigr)+{\mathcal{O}}\bigl( \epsilon^{2}\bigr). $$

It is also possible to derive a corresponding dynamical equation for the two-point correlation function by extending the definition of the effective action along the lines of Buice et al. [20]. However, the lowest-order equation for **C** can be obtained from (5.2). One finds that

6.17 $$\begin{aligned} \frac{dC_{\alpha\beta}}{dt} =&{\mathcal{Q}}_{\alpha\beta}(\mathbf{u})+\sum _{\gamma} \biggl[\frac{\partial\overline{V}_{\alpha}(\mathbf{u})}{\partial u_{\gamma}}C_{\gamma\beta}+ C_{\alpha\gamma}\frac{\partial \overline {V}_{\beta}(\mathbf{u})}{\partial u_{\gamma}} \biggr] \\ =&\sum_{\gamma}w_{\alpha\gamma}F(u_{\gamma})w_{\beta\gamma}+ \sum_{\gamma} \bigl[-\delta_{\alpha, \gamma}+w_{\alpha\gamma }F'(u_{\gamma }) \bigr]C_{\gamma\beta} \\ &{} +\sum_{\gamma} \bigl[-\delta_{\beta, \gamma}+w_{\beta \gamma }F'(u_{\gamma}) \bigr]C_{\alpha\gamma} \\ =&-2C_{\alpha\beta}+\sum_{\gamma}w_{\alpha\gamma}F(u_{\gamma })w_{\beta \gamma} \\ &{}+\sum_{\gamma}F'(u_{\gamma}) [w_{\alpha\gamma }C_{\gamma \beta}+w_{\beta\gamma}C_{\alpha\gamma} ]. \end{aligned}$$

The corrections to mean-field theory for a stochastic hybrid neural network differ significantly from those derived for the Buice et al. master equation [17, 20]. There are two primary sources of such differences. One arises from the fact that the mean equation is in ‘Amari form’ (with the weight matrix outside the nonlinearity). This accounts for all the difference in (6.16) for the mean, which would otherwise be identical to that of Buice et al., and the last term involving *C* in (6.17). The other difference is in the non-homogeneous source term for the *C* equation, which appears as $\sum_{\gamma}w_{\alpha\gamma}F(u_{\gamma })w_{\beta \gamma} $. Whereas the Buice et al. correlations are determined by multiple network motifs (with the lowest order being the direct connection $w_{\alpha\beta}$ from *β* to *α*), our result for the hybrid model indicates that the source term is given by divergent motifs indicating common input from a third population (population *γ* → populations *α*, *β*).

## Discussion

In conclusion, we have constructed a path-integral representation of solutions to a stochastic hybrid neural network, and shown how this provides a unifying framework for carrying out various perturbation schemes for analyzing the stochastic dynamics, namely, large deviations, diffusion approximations, and corrections to mean-field equations. We highlighted the fact that the path-integral action can be expressed in terms of a Hamiltonian, which is given by the Perron eigenvalue of an appropriately defined linear operator. The latter depends on the transition rates and drift terms of the underlying hybrid system. The resulting action is consistent with that obtained using large-deviation theory.

In terms of the theory of stochastic neural networks, our hybrid model extends the neural master equation to include the effects of synaptic currents. In the limit of fast synapses one recovers the neural master equation, which can be viewed as a stochastic version of the ‘Wilson–Cowan’ rate equations (with the weight matrix inside the nonlinearity). On the other hand, in the case of slow synapses, one obtains a stochastic version of the ‘Amari’ rate equations. This leads to significant differences in the corrections to the mean-field equations. Finally, it should be noted that the path-integral formulation presented here can be applied to more general stochastic hybrid systems such as stochastic ion channels, molecular motors, and gene networks [28–32]. Thus one can view our path-integral construction as the hybrid analog of the Doi–Peliti path integral for master equations.
